# Impact of a teat disinfectant based on *Lactococcus cremoris* on the cow milk proteome

**DOI:** 10.1186/s12917-024-04014-x

**Published:** 2024-10-03

**Authors:** Maria Filippa Addis, Elisa Margherita Maffioli, Alessandra Gazzola, Federica Santandrea, Gabriella Tedeschi, Renata Piccinini

**Affiliations:** 1 Department of Veterinary Medicine and Animal Sciences, University of Milan, Lodi, Italy; 2 Laboratory of Animal Infectious Diseases (MiLab), University of Milan, Lodi, Italy; 3 CRC “Innovation for Well-Being and Environment (I-WE)”, University of Milan, Milan, Italy; 4 Istituto Zooprofilattico Sperimentale della Lombardia e dell’Emilia-Romagna, Lodi, 26900 Italy

**Keywords:** Milk proteomics, Teat dipping, Milking routine, Antimicrobial, Immunostimulation, Shotgun proteomics, Cathelicidin

## Abstract

**Background:**

Dairy cow milking practices require cleaning and disinfection of the teat skin before and after milking to ensure the safety and quality of milk and prevent intramammary infections. Antimicrobial proteins of natural origin can be valuable alternatives to traditional disinfectants. In a recent field trial, we demonstrated that a teat dip based on a nisin A-producing *Lactococcus cremoris* (L) had comparable efficacy to conventional iodophor dip (C) in preventing dairy cow mastitis. Here, we present the differential shotgun proteomics investigation of the milk collected during the trial.

**Methods:**

Four groups of quarter milk samples with low (LSCC) and high somatic cell count (HSCC) collected at the beginning (T0) and end (TF) of the trial were analyzed for a total of 28 LSCC (14 LSCC T0 and 14 LSCC TF) and 12 HSCC (6 HSCC T0 and 6 HSCC TF) samples. Milk proteins were digested into peptides, separated by nanoHPLC, and analyzed by tandem mass spectrometry (LC-MS/MS) on an Orbitrap Fusion Tribrid mass spectrometer. The proteins were identified with MaxQuant and interaction networks of the differential proteins were investigated with STRING. The proteomics data have been deposited to the ProteomeXchange Consortium via the PRIDE partner repository with the dataset identifier PXD045030.

**Results:**

In healthy milk (LSCC), we detected 90 and 80 differential proteins at T0 and TF, respectively. At TF, the *Lactococcus* group showed higher levels of antimicrobial proteins. In mastitis milk (HSCC), we detected 88 and 106 differential proteins at T0 and TF, respectively. In the *Lactococcus* group, 14 proteins with antimicrobial and immune defense functions were enriched at TF vs. 4 proteins at T0. Cathelicidins were among the most relevant enriched proteins. Western immunoblotting validation confirmed the differential abundance.

**Conclusions:**

At T0, the proteomic differences observed in healthy milk of the two groups were most likely dependent on physiological variation. On the other hand, antimicrobial and immune defense functions were higher in the milk of cows with mammary gland inflammation of the *Lactococcus*-treated group. Among other factors, the immunostimulatory action of nisin A might be considered as a contributor.

**Supplementary Information:**

The online version contains supplementary material available at 10.1186/s12917-024-04014-x.

## Background

Dairy cow milking practices include cleaning and disinfection of the teat skin before and after milking to ensure the safety and quality of milk and prevent the occurrence of new intramammary infections (IMI) by reducing microbial colonization [1, 2]. This practice is generally performed using chemical disinfectants. However, this might contribute to increasing antibiotic resistance or interfere with the cheesemaking process due to residual antimicrobial action on beneficial microorganisms [3, 4]. Antimicrobial proteins of natural origin, including bacteriocins and essential oils, can be a valuable alternative as active components of teat dipping products [5–7].

A recent study by our group evaluated the efficacy of teat dips based on a *Lactococcus cremoris* strain (*L. cremoris* FT27), as an alternative to conventional iodine-based products. In a three-month field trial, we demonstrated that the teat dips based on *L. cremoris* FT27 had comparable efficacy to a conventional iodophor-based disinfectant in preventing dairy cow mastitis. *L. cremoris* FT27 displayed high bactericidal activity in vitro against the main mastitis pathogens, and the proteomic and genomic analysis revealed that its antimicrobial activity was related to the high production of nisin A [8]. Nisin A is an antimicrobial peptide produced by certain strains of lactococci and is one of the most well-studied and widely used lantibiotics [9, 10]. Its mode of action relies on the disruption of bacterial cell membranes and cell walls, leading to cell death with a duplicate mechanism consisting of pore formation in the bacterial membrane in combination with inhibition of peptidoglycan synthesis [11–14]. Thanks to its natural origin and targeted antimicrobial properties, nisin A can be an attractive alternative to synthetic preservatives for preventing the growth of spoilage and pathogenic microorganisms [15]. Nisin A has been approved as a food preservative by many regulatory agencies, including the U.S. Food and Drug Administration (FDA) and the European Food Safety Authority (EFSA). Nisin A has also been studied for its potential applications in medicine, including the development of novel antibiotics or as an ingredient in wound dressings and other medical products [15–17].

To fully explore these potential applications and address any challenges related to other effects on living organisms upon direct application, dedicated research studies are however needed. Shotgun proteomics is a high-throughput method used for the large-scale identification and quantitation of proteins within a complex biological sample. Among its many advantages, it enables the comprehensive analysis of a large number of proteins from complex mixtures in a single experiment. On the other hand, the number of samples that can be analyzed is limited due to economic and throughput constraints [18]. To investigate the possible changes occurring in the milk upon exposure of the teat to the *Lactococcus*-based teat disinfectant in comparison to the conventional iodophor disinfectant, we selected a subset of milk samples generated in the field trial and characterized them by differential shotgun proteomics followed by functional network analysis.

## Materials and methods

### Teat disinfectant trial

The trial and the teat dip have been described in detail by Gazzola et al. [8]. Briefly, 380 cows from two commercial dairy farms in the Lombardy region (Italy) were enrolled in the study; of these, 182 were treated with the *Lactococcus*-based teat dip and 198 with a commercial iodophor. *L. cremoris* FT27 (deposited in the Agro-Food Microbial culture Collection of the Institute of Sciences of Food Production, CNR, Bari, Italy as ITEM 18332) was used for its ability to produce bacteriocins and for the previously demonstrated antimicrobial activity against food pathogens, as well as against the most important mastitis-causing microorganisms (*Staphylococcus aureus*,* Streptococcus agalactiae*,* Str. uberis*,* Str. dysgalactiae*,* Escherichia coli*,* Enterococcus faecalis*). Quarter milk samples were collected every two weeks for three months for bacteriological analysis and somatic cell counting (SCC). The bacteriological culture was carried out according to the National Mastitis Council guidelines [19]; the colonies, if present, were identified using matrix-assisted laser desorption/ionization time-of-flight mass spectrometry (MALDI-TOF MS) as described previously [20]. The SCC was determined by fluorescent somatic cell counting (Bentley Somacount 150, Bentley Instrument, Chaska, MN, USA). No significant differences were recorded between the two disinfectant groups either in the SCC values or in the bacteriological results. The overall frequency of bacteriologically positive quarters was low and was not related to consistent SCC increases.

## Milk sample selection and preparation

The data obtained during the trial were analyzed to identify matched milk samples collected from the same quarter of the same animal at the beginning (T0) and at the end (TF) of the trial in the two groups: the *Lactococcus*-based (L) and the conventional (C) iodophor-based disinfectant. Four sample categories were defined to include culture-negative low-SCC and high-SCC milk; specifically, low SCC milk (LSCC) was defined as < 15,000 cells/mL, while high SCC milk (HSCC) was defined as > 50,000 cells/mL. Culture-negative samples were selected to avoid biases introduced by different pathogens or by the intramammary infection itself. We identified suitable milk samples collected from the same culture-negative quarters of the same cows at the beginning (T0) and at the end of the study (TF) in the two C and L disinfectant groups. Fourteen and six matched quarter milk samples per disinfectant group were recovered for the LSCC and the HSCC categories, respectively, for a total of 40 samples. The experimental groups and related data are detailed in Table 1. The milk aliquots were retrieved from the frozen sample archive, thawed, and delipidated as described previously [21]. Briefly, the milk was centrifuged at 5000 x g for 15 min at 4 °C. The skim milk was then recovered, centrifuged again at 15,000 x g for 30 min to obtain a casein-depleted supernatant, and subjected to protein quantitation with the Pierce™ Rapid Gold BCA Protein Assay Kit (Thermo Fisher Scientific).

**Table 1:** Quarter milk samples subjected to shotgun proteomics. Acronym, number of samples, somatic cell count (SCC) values in number of cells x 10^3^ per mL of milk, type of dipping product applied, and time of collection, are reported for each sample category

Sample group	*N* (40)	SCC x 10^3^ (mean ± SD)	Teat dipping product	Collection time
LSCC-C T0	7	2.00 ± 1.41	Conventional	Beginning of study
LSCC-L T0	7	1.57 ± 0.79	*L. cremoris*	Beginning of study
LSCC-C TF	7	2.57 ± 2.15	Conventional	End of study
LSCC-L TF	7	5.28 ± 4.35	*L. cremoris*	End of study
HSCC-C T0	3	86 ± 67.00	Conventional	Beginning of study
HSCC-L T0	3	409 ± 212.60	*L. cremoris*	Beginning of study
HSCC-C TF	3	156 ± 25.87	Conventional	End of study
HSCC-L TF	3	594 ± 442.00	*L. cremoris*	End of study

## Shotgun proteomics

The delipidated milk samples were analyzed by a shotgun label-free proteomic approach for the identification and quantification of expressed proteins. Prior to proteolysis, proteins were reduced with 13 mM dithioerythritol (30 min at 55 °C) and alkylated with 26 mM iodoacetamide (20 min at room temperature). Protein digestion was performed using sequence-grade trypsin (Promega) for 16 h at 37 °C using a protein/enzyme ratio of 50:1 [22]. The collected peptides were desalted using Zip-Tip C18 before Mass Spectrometry (MS) analysis. NanoHPLC coupled to MS/MS analysis was performed on a Dionex UltiMate 3000 directly connected to an Orbitrap Fusion Tribrid mass spectrometer (Thermo Fisher Scientific) by a nanoelectrospray ion source. Peptide mixtures were pre-concentrated onto an Acclaim PepMap 100–1000 μm x 2 cm C18 and separated on 75 μm ID × 250 mm EASY-Spray PepMap RSLC C18 column (Thermo Fisher Scientific). The LC gradient was 1% acetonitrile (ACN) in 0.1% formic acid for 10 min, 1–4% ACN in 0.1% formic acid for 6 min, 4–30% ACN in 0.1% formic acid for 147 min, and 30–50% ACN in 0.1% formic for 3 min at a flow rate of 0.3 µL/min. Orbitrap-MS spectra of eluting peptides were collected over an m/z range of 375–1500 at a resolution of 120,000, operating in a data-dependent mode with a cycle time of 3 s between master scans. HCD MS/MS spectra were acquired in Orbitrap at a resolution of 15,000 with a collision energy set at 35% and an isolation window of 1.6 m/z. Dynamic exclusion was set to 60 s. Rejection of + 1 and unassigned charge states were enabled. Database search was conducted against the *Bos taurus* Uniprot sequence database (release on 15 May 2022) with MaxQuant (version 1.6.0.1) software using the following parameters: initial maximum allowed mass deviation of 10 ppm for monoisotopic precursor ions and 0.5 Da for MS/MS peaks, trypsin enzyme specificity, a maximum of 2 missed cleavages, carbamidomethyl cysteine as fixed modification, N-terminal acetylation, methionine oxidation and asparagine/glutamine deamidation as variable modifications. The minimum required peptide length was set to 7 amino acids and the minimum number of unique peptides supporting protein identification was set to 1 [23]. Quantification was performed using the built-in XIC-based label-free quantitation (LFQ) algorithm using fast LFQ. False protein identifications (1%) were estimated by searching MS/MS spectra against the corresponding reversed-sequence (decoy) database.

### Statistical analysis

Statistical analysis was performed using the Perseus software (version 1.5.5.3). Seven and three biological replicates for low SCC and high SCC milk, respectively, each one replicated three times were carried out. Only proteins present and quantified in 71% (low SCC milk) and 100% (high SCC milk) of the repeats were considered as positively identified in a sample and used for statistical analyses. Focusing on specific comparisons (LSCC-L T0 vs. LSCC-C T0, LSCC-L TF vs. LSCC-C TF, HSCC-L T0 vs. HSCC-C T0, and HSCC-L TF vs. HSCC-C TF), proteins were considered differentially abundant if they were present only in one condition or showed significant t-test difference (Student’s T-test p-value ≤ 0.05). Differential proteins were analyzed by STRING: functional protein association networks database (Version 12.0, http://string-db.org/) using the multiple proteins function, with medium confidence (0.400) for the minimum required interaction score, and *Bos taurus* as the organism.

## Western immunoblotting

Cathelicidin (CATHL) abundance was assessed by Western immunoblotting as described previously [24] with minor modifications. Briefly, the milk was thawed, and 5 µl were resuspended in 45 µl of Laemmli Buffer (Sigma-Aldrich, Missouri, USA) and incubated at 100 °C for 5 min in a Thermoblock (Eppendorf, Hamburg, Germany). A total of 10 µl were loaded in each well of AnykD polyacrylamide gels and subjected to SDS-PAGE on a Protean Tetra Cell (Bio-Rad, Hercules, CA). MagicMark molecular weight markers (Thermo Fisher Scientific) and a positive control represented by a high SCC, CATHL-positive, milk sample were loaded in the first and last wells of each gel, respectively. Separated proteins were then transferred to nitrocellulose with the Trans-Blot Turbo Transfer System (BioRad), and after transfer the membranes were checked for quality by reversible Ponceau S staining (Sigma Aldrich). After destaining with water, the membranes were blocked for 10 min with EveryBlot Blocking Buffer (BioRad) and incubated for 1 h with a monoclonal anti-CATHL antibody [25]. The membranes were washed and incubated with HRP-conjugated anti-mouse antibodies (Sigma Aldrich). After washing, the reactivity was visualized with a chemiluminescent substrate (Clarity Western ECL substrate, Bio-Rad). The images were digitalized with the iBright 1500 (Thermo Fisher Scientific).

## Results

### Differential shotgun proteomics of milk

The 40 selected milk samples, summarized in Table 1, were subjected to a shotgun proteomic analysis pipeline with label-free quantitation and 4 differential comparisons were carried out as follows. (A) Low SCC milk from the L and C groups at the beginning of the trial (LSCC-L T0 vs. LSCC-C T0), to assess the treatment-independent differences in the low-SCC milk proteome. (B) Low SCC milk from the L and C groups at the end of the trial (LSCC-L TF vs. LSCC-C TF), to assess the treatment-dependent differences in the low-SCC milk proteome. (C) High SCC milk from the L and C groups at the beginning of the trial (HSCC-L T0 vs. HSCC-C T0), to assess the treatment-independent differences in in the high-SCC milk proteome. (D) High SCC milk from the L and C groups at the end of the trial (HSCC-L TF vs. HSCC-C TF), to assess the treatment-dependent differences in the high-SCC milk proteome. The results are detailed in the Supplementary Dataset, including protein isoforms and peptide identification details, and are described below in detail for each comparison.

*A. LSCC-L T0 vs. LSCC-C T0. Differences between low SCC samples of the two disinfectant groups at the beginning of the trial (treatment-independent).* We identified a total of 281 proteins in LSCC-L T0 and 306 proteins in LSCC-C T0 milk. As a result of their differential analysis, 31 proteins were higher (11) or present only (20) in the *Lactococcus* group (from now higher in L), while 59 proteins were either lower (17) in the L disinfectant group or present only (42) in the C group (from now lower in L) (p-value ≤ 0.05, Student T-test on the 259 common proteins present in 71% of the replicates of all groups).

**Fig. 1:**
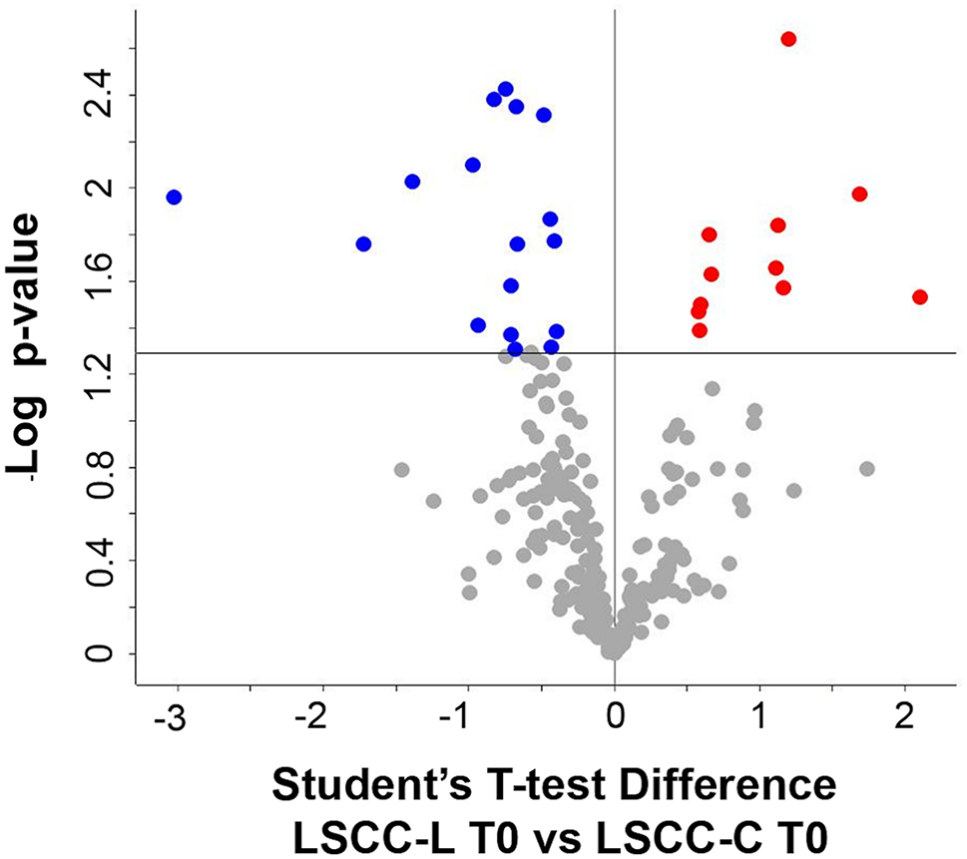
Differential proteins observed in low-SCC milk at the beginning of the trial. The volcano plot illustrates the differential proteins determined using the Student’s T-test (p-value ≤ 0.05). Each protein is represented as a dot and is mapped according to its fold change on the ordinate axis (Y), with the p-value by the T-test on the abscissa (X). The red and blue dots indicate proteins that were higher or lower in L *versus* C, respectively. Grey dots do not meet the FDR criteria

The treatment-independent differences in the low-SCC milk proteome are illustrated in Fig. 1, while differential and unique proteins are summarized in Table 2. Protein identification details can be found in the Supplementary Dataset, sheets 1 and 2.

**Table 2:** List of the differential and unique (u) proteins observed in low somatic cell count milk at the beginning of the trial (LSCC, T0) with their respective student’s T-test difference values. Identification details can be found in the supplementary dataset, sheets 1 and 2

Increased in the *Lactococcus* disinfectant group	T-test difference	Decreased in the *Lactococcus* disinfectant group	T-test difference
Prostaglandin-H2 D-isomerase	2.101	SCGB2A2 protein	-3.027
Lipocalin	1.688	Secretoglobin family 1D member	-1.723
HHIP like 2	1.197	Alkaline phosphatase, tissue-nonspecific isozyme	-1.386
Serpin A3-7	1.166	Pantetheinase	-0.972
Follistatin	1.126	Lactadherin	-0.939
Stromal antigen 1	1.113	Malate dehydrogenase, cytoplasmic	-0.828
DnaJ homolog subfamily C member 3	0.664	Ezrin	-0.745
Peptidyl-prolyl cis-trans isomerase B	0.653	COL18A1 protein	-0.712
Alpha-N-acetylgalactosaminidase	0.595	Cofilin-1	-0.71
Aminopeptidase	0.584	Extracellular matrix protein 1	-0.68
Superoxide dismutase [Cu-Zn]	0.579	Glycosylation-dependent cell adhesion molecule 1	-0.676
ADAM metallopeptidase domain 9	U	Calmodulin	-0.666
Collectin-12	U	Phosphatidylethanolamine-binding protein 1	-0.484
Complement C8 gamma chain	U	Suppressor of tumorigenicity 14 protein homolog	-0.441
Cysteine-rich secretory protein LCCL domain-containing 2	U	Isocitrate dehydrogenase [NADP] cytoplasmic	-0.437
Hsp40 member B9	U	Heat shock protein HSP 90-beta	-0.409
DnaJ homolog subfamily C member 16	U	Actin, cytoplasmic 1	-0.395
Fatty acid synthase	U	14-3-3 protein epsilon	u
F-box protein 38	U	Alpha 1-3-galactosyltransferase	u
Glutathione S-transferase	U	Alpha-actinin-2	u
Granulin	U	Calsyntenin 1	u
Hyaluronidase	U	Cathelicidin-1	u
Ig-like domain-containing protein	U	Ciliary neurotrophic factor receptor	u
Matrix metallopeptidase 24	U	Complement C1s subcomponent	u
NACHT, LRR and PYD domains-containing prot. 3	U	Complement C3	u
NADH-cytochrome b5 reductase 3	U	Complement component 8 subunit beta	u
Phosphoglycerate kinase	U	Copper transport protein ATOX1	u
Protein disulfide-isomerase	U	Cystatin-C	u
Protein S100-B	U	Endoplasmin	u
Protocadherin related 15	U	Ephrin	u
Pyruvate kinase	U	Fructose-bisphosphate aldolase	u
		GDNF family receptor alpha-2	u
		Glyceraldehyde-3-phosphate dehydrogenase	u
		Glycolipid transfer protein	u
		Heat shock 70 kDa protein	u
		Heparanase	u
		Ig-like domain-containing protein	u
		Immunoglobulin M heavy chain	u
		Insulin-like growth factor binding protein 7	u
		Intraflagellar transport protein 56	u
		Lipoprotein lipase	u
		MARCKS-related protein	u
		Metalloproteinase inhibitor 2	u
		Moesin	u
		Myosin	u
		Myosin light chain 3	u
		Myosin regulatory light chain 2	u
		Myosin-1	u
		Myristoylated alanine-rich C-kinase substrate	u
		Peroxiredoxin-4	u
		Progestagen-associated endometrial protein	u
		RAB2A, member RAS onco family	u
		Solute carrier family 22 member 9	u
		Sortilin 1	u
		Thymosin alpha-1	u
		Transcription factor 12	u
		Transketolase	u
		Triosephosphate isomerase	u
		Troponin I1, slow skeletal type	u
		Tubulin alpha-1B chain	u

**Fig. 2:**
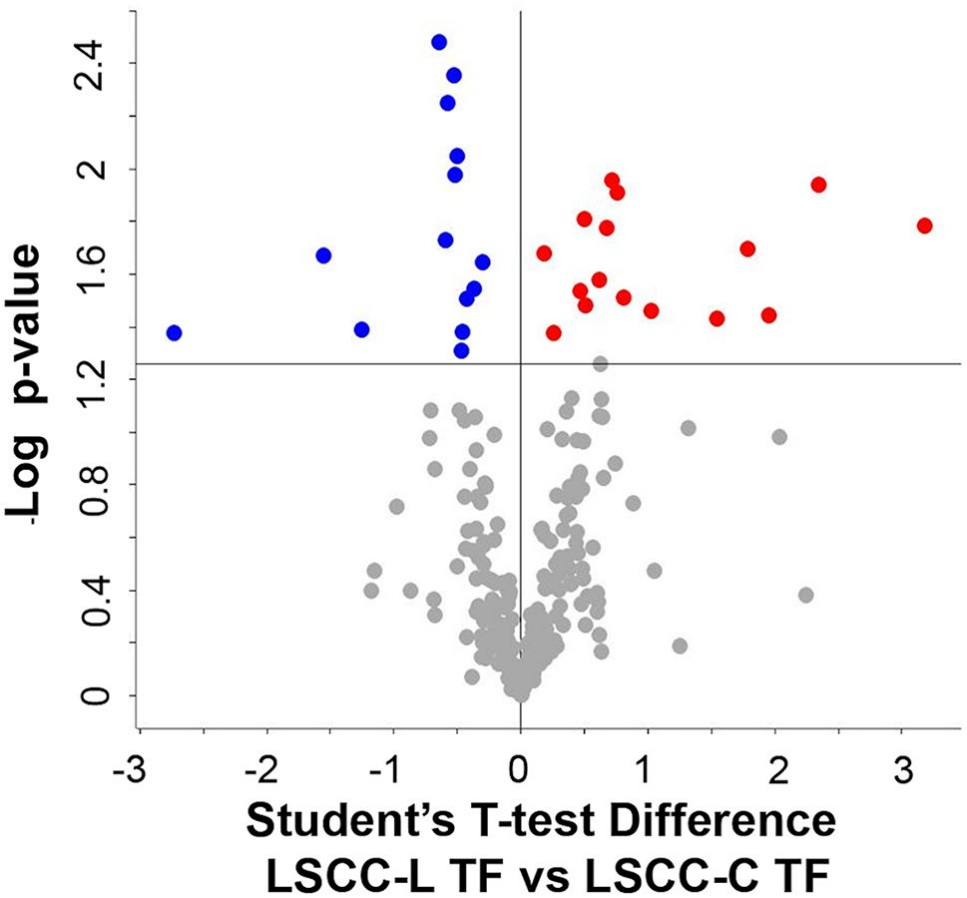
Differential proteins observed in low-SCC milk at the end of the trial. The volcano plot illustrates the differential proteins determined using the Student’s T-test (p-value ≤ 0.05). Each protein is represented as a dot and is mapped according to its fold change on the ordinate axis (Y), with the p-value by the T-test on the abscissa (X). The red and blue dots indicate proteins that were higher or lower in L *versus* C, respectively. Grey dots do not meet the FDR criteria

*B. LSCC-L TF vs. LSCC-C TF. Differences between the two disinfectant groups at the end of the trial (treatment-related).* We identified a total of 285 proteins in LSCC-L TF and 299 proteins in LSCC-C TF milk. As a result of their differential analysis, 34 proteins were higher (18 unique and 16 higher, respectively) and 46 proteins were lower in L (32 unique and 14 lower) (p-value ≤ 0.05, Student T-test on the 266 common proteins present in 71% of the replicates of all groups). The treatment-related differences in the low-SCC milk proteome are illustrated in Fig. 2, while differential and unique proteins are summarized in Table 3. Protein identification details can be found in the Supplementary Dataset, sheets 3 and 4.

**Table 3:** List of the differential and unique (u) proteins observed in low somatic cell count at the end of the trial (LSCC, TF) with their respective student’s T-test difference values. Identification details can be found in the supplementary dataset, sheets 3 and 4

Increased in the *Lactococcus* disinfectant group	T-test difference	Decreased in the *Lactococcus* disinfectant group	T-test difference
Cathelicidin-1	3.177	SCGB2A2 protein	-2.728
Chitinase-3-like protein 1	2.347	Secretoglobin family 1D member	-1.555
Prostaglandin-H2 D-isomerase	1.952	Alkaline phosphatase, tissue-nonspecific isozyme	-1.257
Lipocalin	1.788	Ras-related protein Rab-6B	-0.642
Spermadhesin-1	1.545	Cellular repressor of E1A stimulated 1	-0.597
Unidentified	1.027	Pyridoxal kinase	-0.578
Cathepsin D	0.809	Ras-related protein Rab-11	-0.526
Ig-like domain-containing protein	0.755	Phosphatidylethanolamine-binding protein 1	-0.519
DnaJ homolog subfamily C member 3	0.716	Purine nucleoside phosphorylase	-0.505
Cathepsin S	0.671	Heat shock cognate 71 kDa protein	-0.471
Ephrin-A1	0.613	SH3 domain-binding glutamic acid-rich-like prot. 3	-0.457
Furin	0.51	Ezrin	-0.428
CD59 glycoprotein	0.5	Isocitrate dehydrogenase [NADP] cytoplasmic	-0.366
Lipopolysaccharide-binding protein	0.47	Fructose-bisphosphate aldolase	-0.302
Golgi apparatus protein 1	0.26	Aldose 1-epimerase	u
45 kDa calcium-binding protein	0.186	C1GALT1-specific chaperone 1	u
Centrosomal protein 250	U	ABO, alpha 1-3-N-acetylgalactosaminyltransferase	u
Collectin-43	U	AE binding protein 1	u
Cysteine-rich secretory protein LCCL domain-containing 2	U	Butyrophilin subfamily 1 member A1	u
Hsp40 member B9	U	Calbindin	u
Glyceraldehyde-3-phosphate dehydrogenase	U	Ciliary neurotrophic factor receptor	u
Golgi membrane protein 1	U	Clusterin	u
Granulin	U	Galectin-3-binding protein	u
GRIP and coiled-coil domain containing 2	U	Glucosidase 2 subunit beta	u
Histone H2A	U	Glutathione S-transferase Mu 1	u
Lipoprotein lipase	U	Haptoglobin	u
Lymphocyte cytosolic protein 1	U	Heat shock 70 kDa protein	u
MARCKS-related protein	U	Iduronate 2-sulfatase	u
Metalloproteinase inhibitor 1	U	Ig-like domain-containing protein	u
Metalloproteinase inhibitor 3	U	Inorganic pyrophosphatase	u
Complement C3	U	Insulin-like growth factor binding protein 7	u
Neuromedin-C	U	Isoleucine–tRNA ligase	u
Peroxiredoxin-4	U	Lysosomal protective protein	u
Tetratricopeptide repeat domain 37	U	Myosin regulatory light chain 2	u
		Myosin-1	u
		NACHT, LRR and PYD domains-containing protein 3	u
		Pantetheinase	u
		Phospholipid transfer protein	u
		Pyruvate kinase	u
		Receptor (Chemosensory) transporter protein 4	u
		Sortilin 1	u
		Syndecan-2	u
		Thioredoxin	u
		Transcription Factor 12	u
		Transketolase	u
		Vesicle amine transport 1	u

**Fig. 3:**
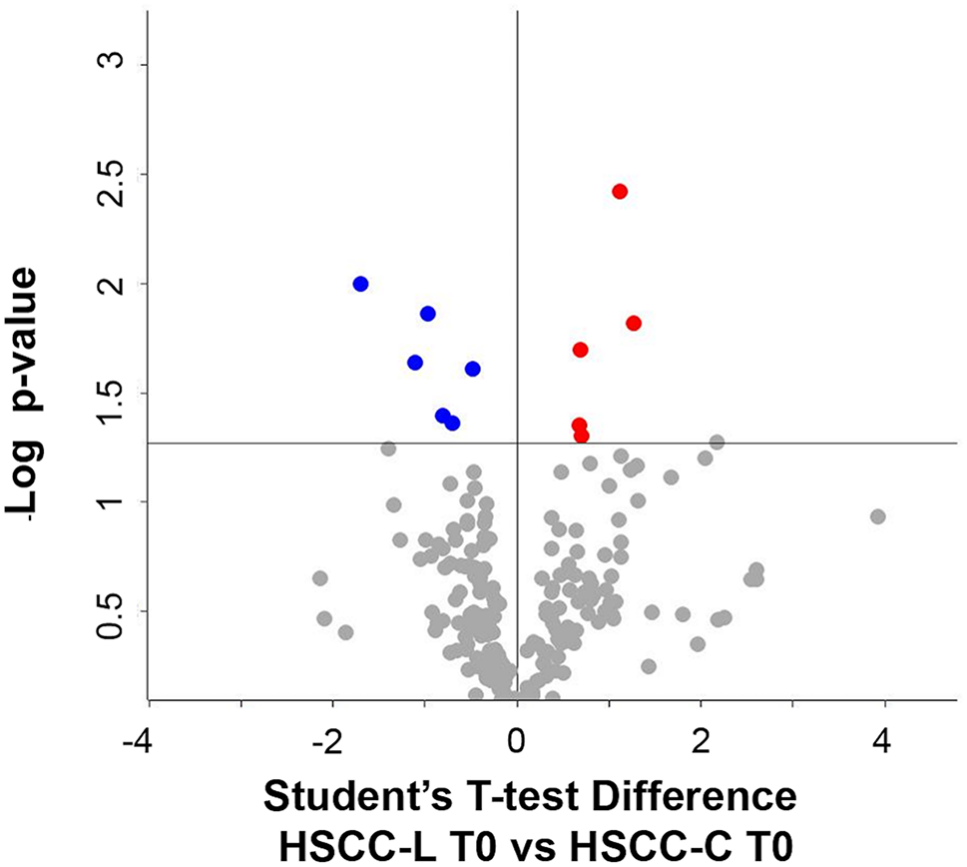
Differential proteins observed in high-SCC milk at the beginning of the trial. The volcano plot illustrates the differential proteins determined using the Student’s T-test (p-value ≤ 0.05). Each protein is represented as a dot and is mapped according to its fold change on the ordinate axis (Y), with the p-value by the T-test on the abscissa (X). The red and blue dots indicate proteins that were higher or lower in L *versus* C, respectively. Grey dots do not meet the FDR criteria

*C. HSCC-L T0 vs. HSCC-C T0. Differences between high SCC samples of the two disinfectant groups at the beginning of the trial (treatment-independent).* We identified a total of 287 proteins in HSCC-L T0 and 264 proteins in HSCC-C T0 and milk. As a result of their differential analysis, 54 proteins were higher (47 unique and 7 higher) and 35 proteins were lower in L (28 unique and 7 lower, respectively) (p-value ≤ 0.05, Student T-test on the 236 common proteins present in 100% of the replicates of all groups). The treatment-independent differences in the high-SCC milk proteome are illustrated in Fig. 3, while differential and unique proteins are summarized in Table 4. Protein identification details can be found in the Supplementary Dataset, sheets 5 and 6.

**Table 4:** List of the differential and unique (u) proteins observed in high somatic cell count milk at the beginning of the trial (HSCC, T0) with their respective student’s T-test difference values. Identification details can be found in the supplementary dataset, sheets 5 and 6

Increased in the Lactococcus disinfectant group	T-test difference	Decreased in the Lactococcus disinfectant group	T-test difference
Mucin-2	5.094	Major facilitator superfamily domain cont. 4B	-4.289
Septin-5	2.766	5’-nucleotidase	-1.705
Transforming growth factor beta receptor 3	1.268	Nucleoside diphosphate kinase	-1.113
Cystatin-C	1.119	ATP-binding cassette sub-family G member 2	-0.970
Transforming protein RhoA	0.694	Aldose 1-epimerase	-0.810
Protein kinase C-binding protein NELL2	0.692	Ras-related protein Rab-11	-0.703
Adhesion G protein-coupled receptor G1	0.679	Fibronectin	-0.482
ACD shelterin complex sub. and telomerase recruitment factor	u	Ribonuclease	u
Adenosylhomocysteinase	u	Butyrophilin subfamily 1 member A1	u
Biglycan	u	EF-hand calcium binding domain 14	u
C1GALT1-specific chaperone 1	u	Fructose-bisphosphate aldolase	u
Cathelicidin-3	u	Glutathione S-transferase Mu 1	u
Cathelicidin-6	u	Glypican-1	u
Cathelicidin-7	u	Heparanase	u
CD81 antigen	u	Histone-lysine N-methyltransferase	u
Chitinase-3-like protein 1	u	Lipoprotein lipase	u
Clusterin	u	Macrophage migration inhibitory factor	u
Coactosin-like protein	u	Pantetheinase	u
Collectin-43	u	Protein phosphatase 1 regulatory subunit 7	u
Complement C8 gamma chain	u	Protocadherin related 15	u
Fatty acid-binding protein, epidermal	u	Putative phospholipase B-like 2	u
Granulin	u	Selenium-binding protein 1	u
Growth/differentiation factor 8	u	G protein-coupled receptor class C group 5 m. B	u
Heat shock protein HSP 90-alpha	u	Glycogen synthase kinase-3 beta	u
Histidine-rich glycoprotein	u	IgM Heavy chain	u
Histidine-rich glycoprotein	u	Coiled-coil domain containing 191	u
Histone H2B	u	Ig-like domain-containing protein	u
Ig-like domain-containing protein	u	MFS profile domain-containing protein	u
Jacalin-type lectin domain-containing protein	u	IQ motif containing with AAA domain 1 like	u
Lipocalin	u	Mitogen-activated protein kinase kinase kinase 3	u
Lymphocyte cytosolic protein 1	u	NTN4 protein	u
Malate dehydrogenase, mitochondrial	u	PP1201 protein	u
MARCKS-related protein	u	Tumor protein p53 binding protein 2	u
Membrane cofactor protein	u	Vacuolar protein sorting 13 homolog D	u
Metalloproteinase inhibitor 1	u	Exportin 4	u
Moesin	u		u
Myristoylated alanine-rich C-kinase substrate	u		u
NAD(P)(+)--arginine ADP-ribosyltransferase	u		u
Neuromedin-C	u		u
Peroxiredoxin-4	u		u
Phosphoglycerate kinase	u		u
Protein disulfide-isomerase A4	u		u
Protein disulfide-isomerase A6	u		u
Prothymosin alpha	u		u
Protocadherin gamma subfamily A, 5	u		u
Ras-related protein Rab-6B	u		u
Serine protease 23	u		u
Serine protease 8	u		u
Serpin A3-7	u		u
Serum amyloid A protein	u		u
Syndecan-2	u		u
Transcription Factor 12	u		u
Transforming growth factor beta-2	u		u
Vimentin	u		u

**Fig. 4:**
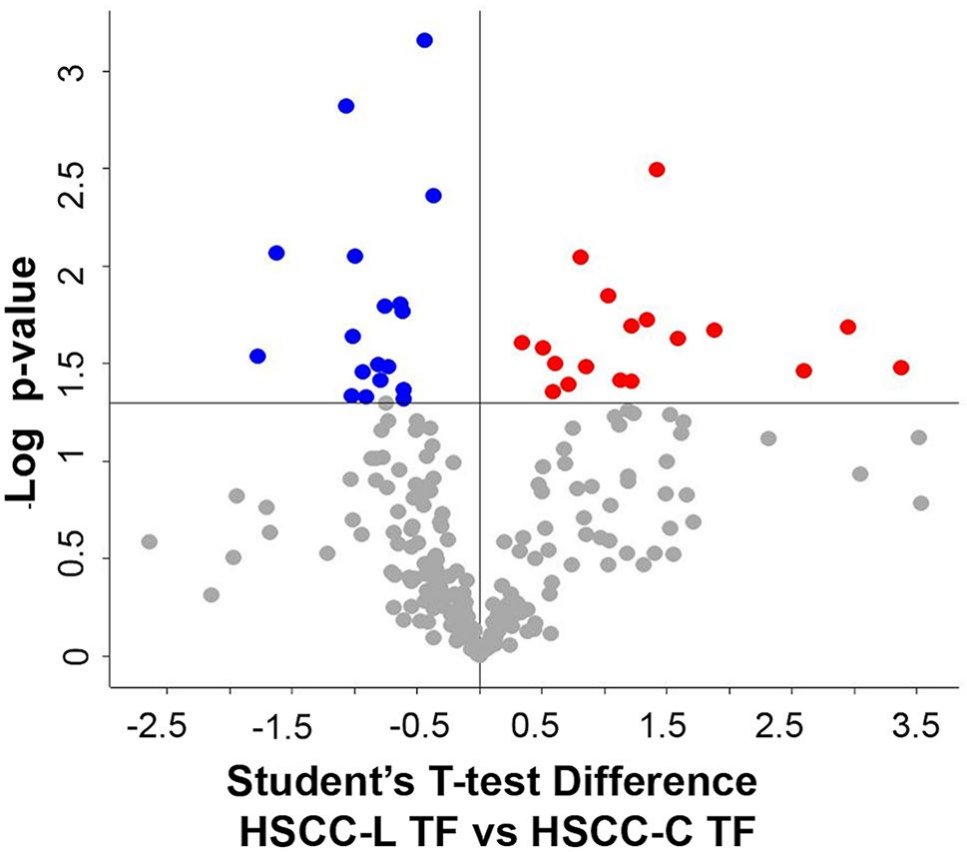
Differential proteins observed in high-SCC milk at the end of the trial. The volcano plot illustrates the differential proteins determined using the Student’s T-test (p-value ≤ 0.05). Each protein is represented as a dot and is mapped according to its fold change on the ordinate axis (Y), with the p-value by the T-test on the abscissa (X). The red and blue dots indicate proteins that were higher or lower in L *versus* C, respectively. Grey dots do not meet the FDR criteria

*D. HSCC-L TF vs. HSCC-C TF. Differences between the two disinfectant groups at the end of the trial (treatment-dependent).* We identified a total of 292 proteins in HSCC-L TF and 263 proteins in HSCC-C TF milk. As a result of their differential analysis, 67 proteins were higher (49 unique and 18 higher) and 39 proteins were lower in L (21 unique and 18 lower, respectively) (p-value ≤ 0.05, Student T-test on the 242 common proteins present in 100% of the replicates of all groups. The treatment-related differences in the high-SCC milk proteome are illustrated in Fig. 4, while differential and unique proteins are summarized in Table 5. Protein identification details can be found in the Supplementary Dataset, sheets 7 and 8.

**Table 5:** List of the differential and unique (u) proteins observed in high somatic cell count milk at the end of the trial (HSCC, TF) with their respective student’s T-test difference values. Identification details can be found in the supplementary dataset, sheets 7 and 8

Increased in the *Lactococcus* disinfectant group	T-test difference	Decreased in the *Lactococcus* disinfectant group	T-test difference
Biglycan	3.379	Fatty acid-binding protein, heart	-1.777
Lipocalin	2.949	Ceruloplasmin	-1.622
Mucin-2	2.593	Dystroglycan	-1.063
Serum amyloid A protein	1.878	Selenium-binding protein 1	-1.019
Neuromedin-C	1.586	Procathepsin L	-1.015
Apolipoprotein C-III	1.421	Mucin-15	-0.993
SH3 domain-binding glutamic acid-rich-like protein 3	1.336	Nucleotide exchange factor SIL1	-0.932
Elongation factor 1-alpha	1.217	Multiple inositol polyphosphate phosphatase 1	-0.906
Alpha-enolase	1.213	Folate receptor alpha	-0.807
Phosphoglycerate mutase 1	1.127	Renin receptor	-0.790
Lactotransferrin	1.032	Cystatin E/M	-0.756
Transforming growth factor beta receptor 3	0.851	Phosphatidylethanolamine-binding protein 1	-0.730
Beta-2-microglobulin	0.809	Thioredoxin domain containing 5	-0.635
Transforming growth factor beta-2	0.708	45 kDa calcium-binding protein	-0.614
Copper transport protein ATOX1	0.604	Peptidyl-prolyl cis-trans isomerase A	-0.610
Ephrin-A1	0.586	Centrosomal protein 250	-0.608
Cysteine-rich secretory protein 2	0.507	Synaptobrevin homolog YKT6	-0.440
Transforming protein RhoA	0.336	Selenoprotein M	-0.371
14-3-3 protein beta/alpha	u	Adhesion G protein-coupled receptor G1	u
ACD shelterin complex subunit and telomerase recruitment factor	u	Alkaline phosphatase, tissue-nonspecific isozyme	u
Actin	u	Anterior gradient 2, protein disulphide isomerase family member	u
Adenosylhomocysteinase	u	Calmodulin	u
Aminopeptidase	u	Calsyntenin 1	u
C2H2-type domain-containing protein	u	Fc fragment of IgA and IgM receptor	u
Cartilage acidic protein 1	u	Furin	u
Cathelicidin-1	u	Glucosidase 2 subunit beta	u
Cathelicidin-3	u	Hypoxia up-regulated 1	u
Cathelicidin-4	u	Ig-like domain-containing protein	u
Cathelicidin-6	u	Insulin-like growth factor binding protein 7	u
CD320 antigen	u	Laminin subunit beta 1	u
CD81 antigen	u	Macrophage migration inhibitory factor	u
Chitinase-3-like protein 1	u	Malate dehydrogenase, mitochondrial	u
Clusterin	u	MARCKS-related protein	u
Coactosin-like protein	u	Mucin 20, cell surface associated	u
Complement C3	u	Pantetheinase	u
Complement C8 gamma chain	u	PP1201 protein	u
Cysteine-rich secretory protein LCCL domain-containing 2	u	Syndecan-2	u
Dickkopf WNT signaling pathway inhibitor 3	u	Thioredoxin	u
Endoplasmin	u	TNF receptor superfamily member 21	u
Fatty acid synthase	u		
Fatty acid-binding protein, adipocyte	u		
Fibulin 2	u		
Golgi membrane protein 1	u		
Histidine-rich glycoprotein	u		
Histone	u		
Ig-like domain-containing protein	u		
Immunoglobulin E heavy chain	u		
Jacalin-type lectin domain-containing protein	u		
Membrane anchored junction protein	u		
Metalloproteinase inhibitor 3	u		
Multimerin 2	u		
Myristoylated alanine-rich C-kinase substrate	u		
N-acetylglucosamine-1-phosphotransferase subunit gamma	u		
Peptidoglycan recognition protein 1	u		
Phospholipid transfer protein	u		
Protein disulfide-isomerase A4	u		
Protein NDRG1	u		
Putative phospholipase B-like 2	u		
Pyruvate kinase	u		
Ras-related protein Rab-6B	u		
Serine protease 22	u		
Serine protease 23	u		
Serine protease 8	u		
Thymosin beta-4	u		
Transcription factor 12	u		
Vimentin	u		

## Functional analysis of the differential proteins

The differential proteins detected at the beginning (T0) and end (TF) of the trial were subjected to protein-protein network analysis by STRING to gather information on their biological functions and networks.

**Fig. 5:**
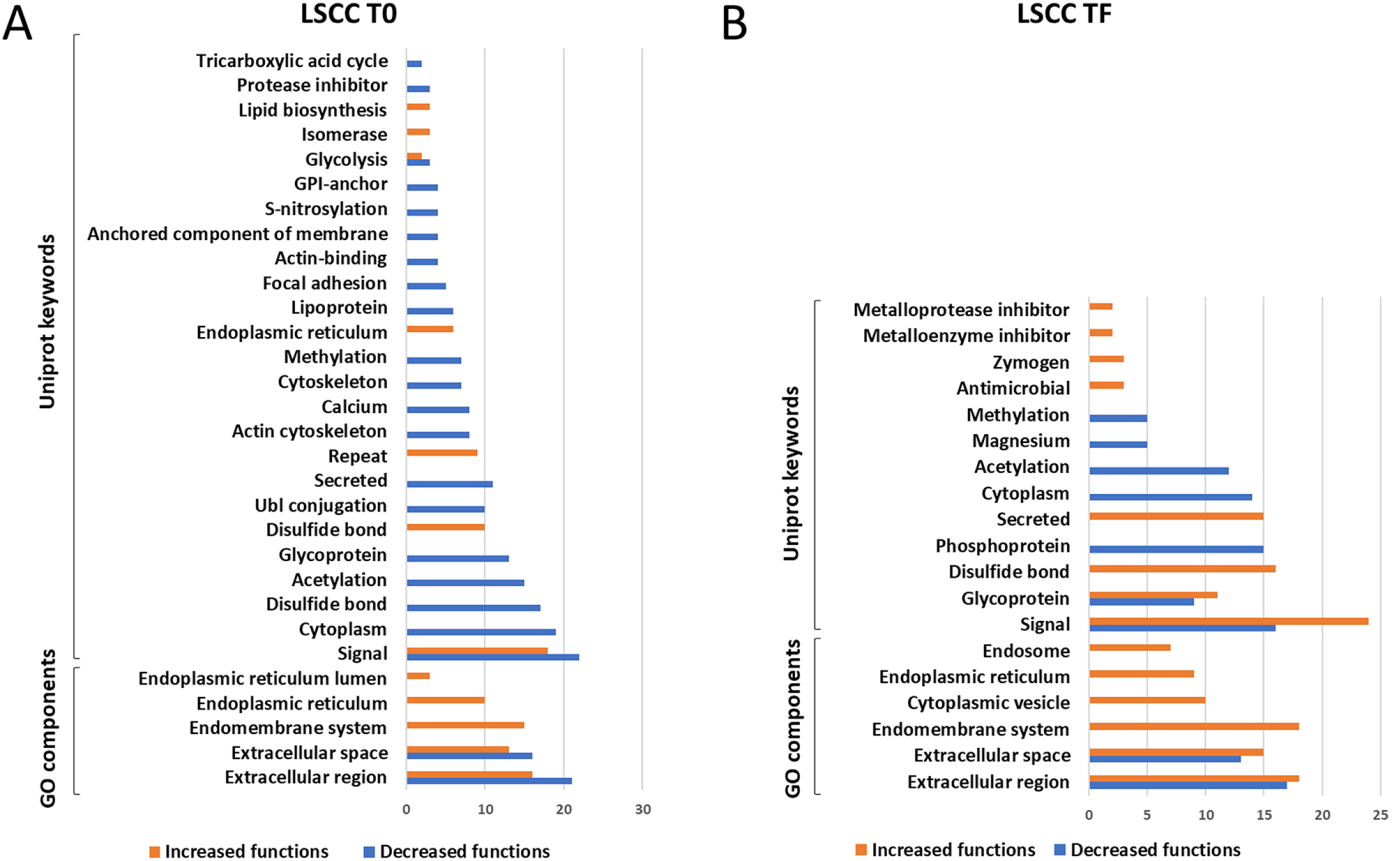
Significant shared categories in low somatic cell count milk. The graph illustrates the proteins increased (orange) or decreased (blue) in the *Lactococcus* disinfectant group compared to the conventional iodophor group at the beginning (**a**, LSCC T0) and at the end (**b**, LSCC TF) of the trial. The bars indicate the number of significantly enriched terms in the respective categories for each disinfectant group. Detailed GO terms, protein IDs, and statistical information can be found in the Supplementary Dataset

*LSCC milk.* All the 90 differential proteins found in LSCC milk at the beginning of the trial (31 increased and 59 decreased, respectively) belonged to biosynthetic metabolic pathways, as indicated by the significance of the Uniprot keywords glycolysis, lipid biosynthesis, tricarboxylic acid cycle, signal, endoplasmic reticulum (Supplementary Dataset, sheets 9 and 10). The protein networks are illustrated in Supplementary File 1, Figures S1 and S2. Immune defense or antimicrobial processes were not significant. The category gene ontology (GO) components was shared between the two disinfectant groups. The terms included in this category are illustrated in Fig. 5A together with the significant Uniprot keywords for the two sample groups. Based on these results, the differences between healthy quarters at the beginning of the trial are dependent on physiological variation among animals, as expected.

Of the total 80 differential proteins observed at the end of the trial, 34 were higher and 46 were lower in L vs. C, respectively. Upon network and gene ontology analysis by STRING, the formers were involved in 4 significantly enriched categories and 10 significant terms, while the latter were involved in 5 significantly enriched categories and 15 significant terms. The results are detailed in the Supplementary Dataset, sheets 11 and 12. The protein networks are illustrated in Supplementary File 1, Figures S3 and S4A. The only significant shared category was GO components. The terms included in this category are illustrated in Fig. 5B together with the significant Uniprot keywords. Notably, the Uniprot Keyword Antimicrobial was significant for the proteins increased in the *Lactococcus* group and involved 3 proteins: chitinase-3-like protein 1, lipopolysaccharide-binding protein, and CATHL 1. These are highlighted within the protein network in Supplementary File 1, Figure S4B.

**Fig. 6:**
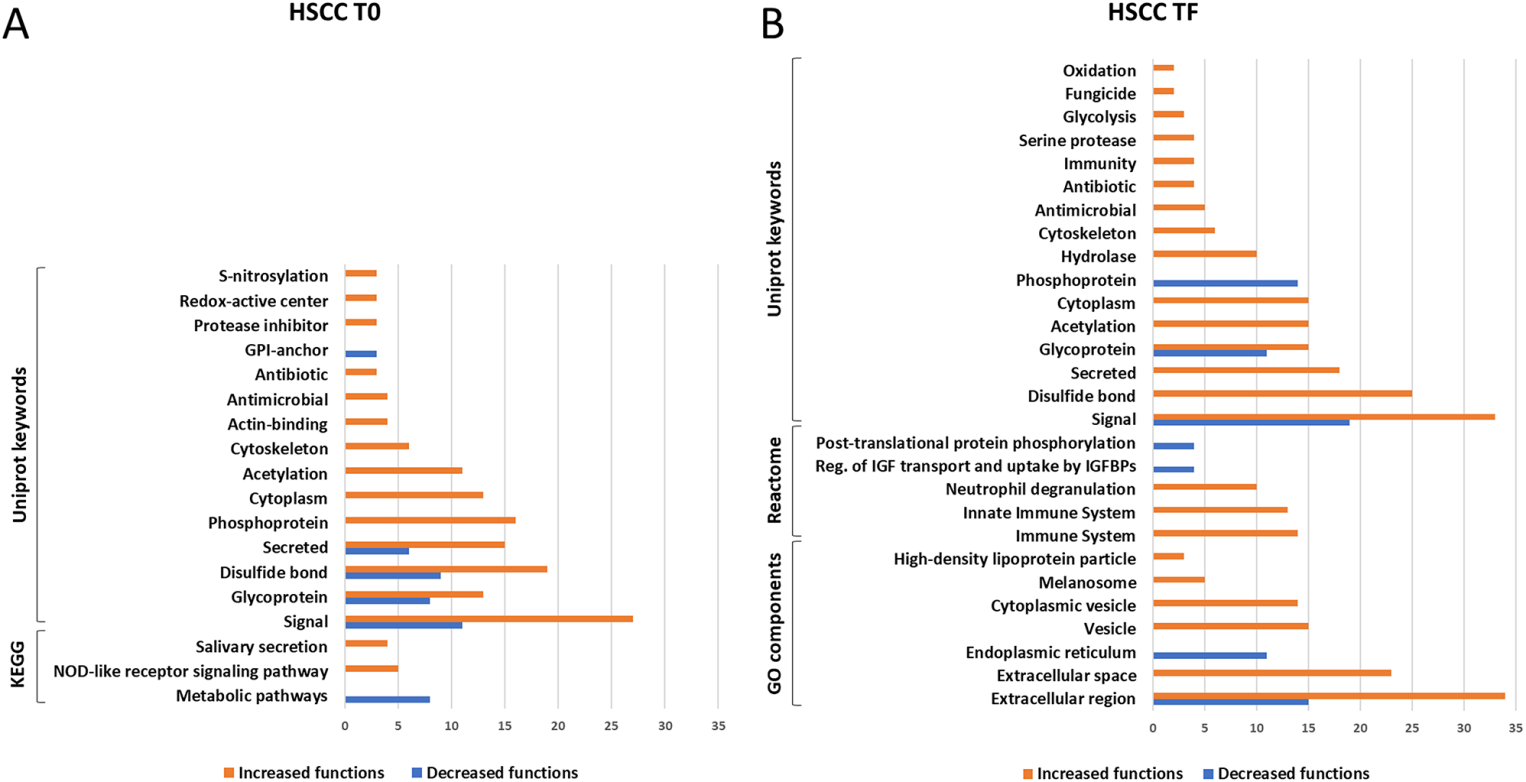
Significant shared categories in high somatic cell count milk. The graph illustrates the proteins increased (orange) or decreased (blue) in the *Lactococcus* disinfectant group compared to the conventional iodophor group at the beginning (A, HSCC T0) and at the end (B, HSCC TF) of the trial. The bars indicate the number of significantly enriched terms in the respective categories for each disinfectant group. Detailed GO terms, protein IDs, and statistical information can be found in the Supplementary Dataset

*HSCC milk.* Of the total 89 differential proteins found in HSCC milk at T0, the 35 proteins decreased in the L group were significantly matched only to Kyoto Encyclopedia of Genes and Genomes (KEGG) metabolic pathways while the 54 proteins increased in the L group were significantly involved in 3 categories and 15 terms. The results are detailed in the Supplementary Dataset, sheets 13 and 14, while the protein networks are illustrated in Supplementary File 1, Figures S5 and S6A. The terms included in the shared KEGG category are compared in Fig. 6A together with the Uniprot keywords. The Uniprot Keywords Antibiotic and Antimicrobial were already enriched in the L group and involved 4 proteins: chitinase-3-like protein 1 and CATHL 3, CATHL 6, and CATHL 7. These are highlighted within the protein network in Supplementary File 1, Figure S6B.

Of the 106 differential proteins found at the end of the trial in HSCC milk, 39 were significantly lower and 67 were higher in L vs. C. Upon network and gene ontology analysis by STRING, the proteins lower in L than C were involved only in 2 categories and 4 terms, while those higher in L than C were involved in 6 categories and 38 significant terms. The results are detailed in the Supplementary Dataset, sheets 15 and 16. The protein networks are illustrated in Supplementary File 1, Figures S7 and S8A. Two significant categories were shared: GO components and Reactome. The terms included in these categories are comparatively illustrated in Fig. 6B together with the Uniprot keywords. Antibiotic, antimicrobial, and immune defense functions were significantly related only to the proteins enriched in the L group and included 14 proteins, four Uniprot Keywords, and three Reactome Pathways. The Uniprot Keywords and the related increased proteins were the following: Fungicide (2 proteins: peptidoglycan recognition protein 1 and CATHL 6), Immunity (4 proteins: lactotransferrin, peptidoglycan recognition protein 1, beta-2-microglobulin, and CD81), Antibiotic (4 proteins: lactotransferrin, peptidoglycan recognition protein 1, CATHL 1, CATHL 6), Antimicrobial (5 proteins: lactotransferrin, chitinase-3-like protein 1, peptidoglycan recognition protein 1, CATHL 1, CATHL 6). The Reactome terms were the following: Neutrophil degranulation (10 proteins: lactotransferrin, peptidoglycan recognition protein 1, transforming protein RhoA, beta-2-microglobulin, cysteine-rich secretory protein 2 and 3, coactosin-like protein 1, cysteine-rich secretory protein LCCL domain-containing 2, phosphoglycerate mutase 1, pyruvate kinase, and elongation factor 1-alpha), Innate immune system (13 proteins: lactotransferrin, peptidoglycan recognition protein 1, endoplasmin, transforming protein RhoA, beta-2-microglobulin, cysteine-rich secretory protein 3, coactosin-like protein 1, cysteine-rich secretory protein LCCL domain-containing 2, phosphoglycerate mutase 1, pyruvate kinase, CD81, elongation factor 1-alpha, complement C8 gamma chain), and Immune system (14 proteins: lactotransferrin, peptidoglycan recognition protein 1, endoplasmin, transforming protein RhoA, beta-2-microglobulin, cysteine-rich secretory protein 2 and 3, coactosin-like-protein 1, phosphoglycerate mutase 1, pyruvate kinase, CD81, aminopeptidase, elongation factor 1-alpha, complement C8 gamma chain). These are highlighted within the protein network in Supplementary File 1, Figure S8B.

### Western immunoblotting validation

**Fig. 7:**
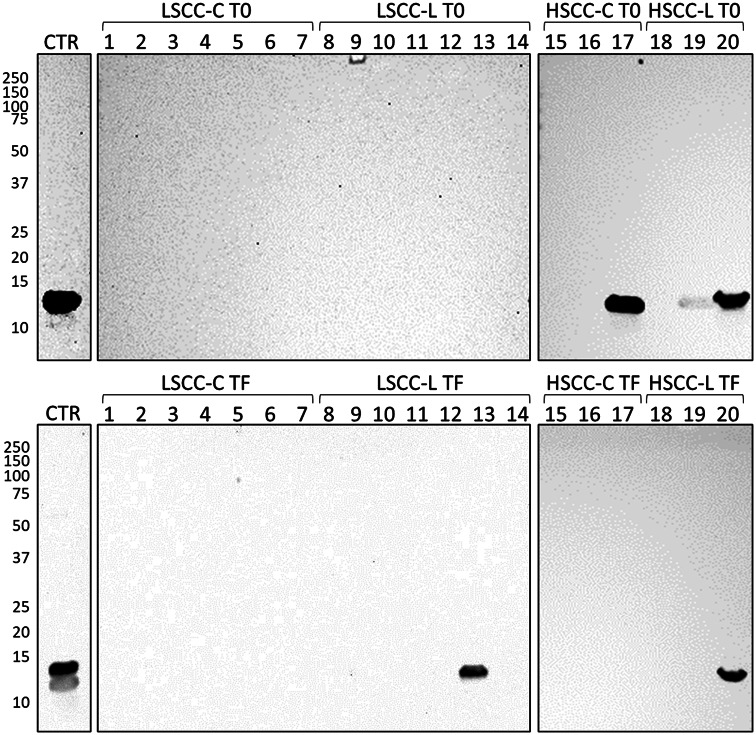
Western immunoblotting of all milk samples with anti-cathelicidin antibodies. CTR, positive control. Molecular weight markers in kDa are indicated on the left. Top images: samples at the beginning of the study (T0); bottom images, samples at the end of the study (TF). LSCC-C, low somatic cell count, conventional iodophor disinfectant group. LSCC-L, low somatic cell count, *Lactococcus* disinfectant group. HSCC-C, high somatic cell count, conventional iodophor disinfectant group. HSCC-L, high somatic cell count, *Lactococcus* disinfectant group. The numbers indicate the lanes in the SDS-PAGE gels; each number corresponds to a different sample. The original Western Immunoblotting images are reported in Supplementary File 2

As expected, a higher number of CATHL isoforms were identified in HSCC than in LSCC milk, consistently with a higher abundance of these proteins in the former group. However, while CATHLs were detected in both *Lactococcus* and conventional iodophor groups at the beginning of the study, their abundance was higher in the *Lactococcus* groups, both LSCC and HSCC, at the end of the study. To validate these findings, we performed western immunoblotting for CATHL. The results are illustrated in Fig. 7. The original Western Immunoblotting images used to assemble Fig. 7 are reported in Supplementary file 2.

## Discussion

Teat cleaning and disinfection represent the basis of hygienic milking aimed at preserving food safety and animal health [26, 27]. This practice, however, is generally performed using disinfectants that might contribute to increasing antibiotic resistance as well as interfere with the cheesemaking process [3]. Antimicrobial substances of natural origin, including bacteriocins and essential oils, can be a valuable alternative as active components [5–7]. However, a sensitive analytical approach is needed to uncover possible changes occurring in the milk proteome following teat and udder exposure to cleaning and disinfection products. To this aim, we selected a sample subset from a field trial comparing conventional and *Lactococcus*-based teat disinfectants and carried out an in-depth differential shotgun proteomics study on LSCC milk (healthy animals) and HSCC milk (animals with subclinical mastitis).

Selecting LSCC milk from the animals of both disinfectant groups that remained healthy throughout the trial was not an issue, as most of the cows’ quarters were not infected. For the same reason, finding cows with HSCC throughout the trial was a challenge. A further constraint was the need to include only milk from quarters with negative bacteriological culture throughout the trial to limit the biases introduced by bacterial infection and avoid pathogen-specific changes in the milk proteome [28, 29]. We could retrieve only six quarter-matched samples for each treatment group with these characteristics. Therefore, HSCC samples were less numerous and more heterogeneous in terms of mean SCC values, and this may represent a limitation of our study concerning HSCC milk.

First, we assessed the differences in the milk proteomes of the cows assigned to the two disinfectant groups at the beginning of the trial (T0, treatment-independent). As expected, most of the differential milk proteins detected at T0 were involved in metabolic pathways and were likely dependent on physiological variability among animals. Indeed, these were significantly associated with annotated Uniprot keywords related to milk production and secretion, including lipid biosynthesis, glycolysis, lipoprotein, endoplasmic reticulum, cytoskeleton, calcium, cell signaling, as well as with the GO components endoplasmic reticulum, endomembrane system, extracellular space, and extracellular region. None of the differential proteins was involved in pathological processes.

In LSCC milk at the end of the trial (TF, treatment-dependent), the differential proteins were also involved in physiological milk production and secretion pathways. However, an increase in antimicrobial proteins (3 out of 34 differential proteins) was detected in the L group, specifically chitinase-3-like protein 1, lipopolysaccharide-binding protein, and CATHL1. All these proteins are involved in the immune response of ruminants against pathogens and are increased in the milk during mastitis [30].

Concerning HSCC milk at T0, similar observations were made. However, the quarters assigned to the HSCC-L group at the beginning of the trial already displayed higher levels of four antimicrobial proteins compared to the quarters assigned to the HSCC-C group, namely chitinase-3-like protein 1 and CATHL3, CATHL6, CATHL7. Chitinase-3-like protein 1 participates in the innate immune response to microbial pathogens at the site of invasion [31]; its increase has been reported in the milk of cows and buffaloes with mastitis [32, 33]. CATHLs are well-known antimicrobial proteins that play a major role in the immune defense of ruminants [34, 35]. Their increased abundance in the presence of mastitis has been reported in the milk of most dairy species [30]. The presence of CATHLs in HSCC milk at the beginning of the study was confirmed by western immunoblotting. The differences detected in HSCC milk at the beginning of the trial (T0), although minor, should be taken into account when assessing the treatment-dependent differences at its end (TF).

At the end of the trial, the differences between HSCC milk of the two disinfectant groups became more relevant. Once again, all the 39 proteins decreased in the L group were involved in physiological milk production and secretion pathways, while 17 of the 67 proteins increased in the L group were involved in antimicrobial and immune defense pathways and included CATHL1, 3, 4, 6, lactotransferrin, chitinase-3-like protein 1, complement fractions, serum amyloid A, histones, and vimentin. All these proteins have been reported to increase in the milk of ruminants during mastitis and are mainly associated with the innate immune response [30, 36–41]. Positive bands corresponding to the molecular weight of CATHL isoforms were detected by western immunoblotting in several samples belonging to the groups with higher abundance according to mass spectrometry, although not in all of them. This might be related to aspects including different abundance among samples, different isoform specificity or lower sensitivity of the antibody by western immunoblotting compared to ELISA, the platform for which it was developed and validated [24].

As the field trial demonstrated that the efficacy of the two disinfectants in preventing mastitis is comparable [8], the observation of higher levels of antimicrobial and innate immune defense proteins in the *Lactococcus*-disinfectant group opens to several considerations. First, these may be related to the slightly higher SCC in the samples of the HSCC-L group, and therefore to the greater presence of neutrophils and their proteins, and not due to the *L. cremoris* teat dip [42]. Further, different etiological agents might have caused undetected IMIs in the two animal groups, leading to differences in the mastitic milk proteome [29, 43, 44]. As these were negative to bacteriological culture, we have no information in this respect [45].

All this considered, the selective immunostimulatory activity of nisin might also be contemplated as a contributor. Nisin is the effector responsible for the antimicrobial activity of the *Lactococcus*-based disinfectant, and the strain selected for its preparation is a high nisin producer [8]. Similarly to other antimicrobial peptides of natural origin, it has been shown that the spectrum of biological activities of nisin goes far beyond its antibacterial properties, including still uncharacterized and incompletely understood immunomodulatory activities [10, 46, 47]. The protective effect of nisin in bacterial infection would not be limited to its direct bactericidal action but possibly involve the additional harmonization of the host immune response towards the resolution of the infection [10]. Immunostimulatory effects have also been reported for lactobacilli as live probiotics in teat dips and intramammary infusions [48–50].

## Conclusions

In conclusion, the *Lactococcus*-based disinfectant was comparable to the commercial disinfectant in terms of IMI and SCC in the field trial. Based on shotgun proteomic analysis, the number of differential milk proteins in healthy quarters was comparable at the beginning and the end of the study and the proteins showing differential abundances were mainly involved in physiological processes, indicating that the observed changes were most likely dependent on individual differences between animals. Interestingly, however, the antimicrobial function was increased at the end of the trial in the *Lactococcus-*disinfectant group. Finally, in animals with an underlying mammary gland inflammation, the increase in antibiotic, antimicrobial, and immune defense functions observed in the *Lactococcus*-disinfectant group was more pronounced and involved a higher number of proteins. Individual variables including differences in the underlying inflammatory status of each animal within HSCC groups, and heterogeneity within and among sample groups, are a limitation of this study, also in consideration of the reduced number of samples that could be analyzed for the HSCC category. Nevertheless, an immunostimulatory effect of nisin, the effector responsible for the antimicrobial action of *L. cremoris*, deserves consideration as a contributor of the observed results.

## Electronic supplementary material

Below is the link to the electronic supplementary material.

Supplementary Material 1

Supplementary Material 2

Supplementary Material 3

## Data Availability

The dataset supporting the conclusions of this article have been deposited to the ProteomeXchange Consortium via the PRIDE partner repository (https://www.ebi.ac.uk/pride/archive?sortDirection=DESC&page=0&pageSize=20) with the dataset identifier PXD045030.

## Abbreviations

L: Lactococcus-based teat disinfectant
C: Conventional iodophor-based teat disinfectant
T0: Time point zero
TF: Final time point
SCC: Somatic cell count
LSCC: Low somatic cell count
HSCC: High somatic cell count
LC-MS/MS: Liquid chromatography-tandem mass spectrometry
IMI: Intramammary infection
MALDI-TOF MS: Matrix-assisted laser desorption/ionization time-of-flight mass spectrometry
ACN: Acetonitrile
HCD: Higher-energy collisional dissociation
LFQ: Label-free quantitation
CATHL: Cathelicidin
GO: Gene ontology
KEGG: Kyoto Encyclopedia of Genes and Genomes
