# Supplementary material for: Impact of a teat disinfectant based on Lactococcus cremoris on the cow milk proteome
Source: BMC Vet Res. 2024 Oct 3;20:447. doi: 10.1186/s12917-024-04014-x (PMC11448288; doi:10.1186/s12917-024-04014-x)
Supplement: Supplementary file 2 — Supplementary Material 2 [file 12917_2024_4014_MOESM2_ESM.pdf]

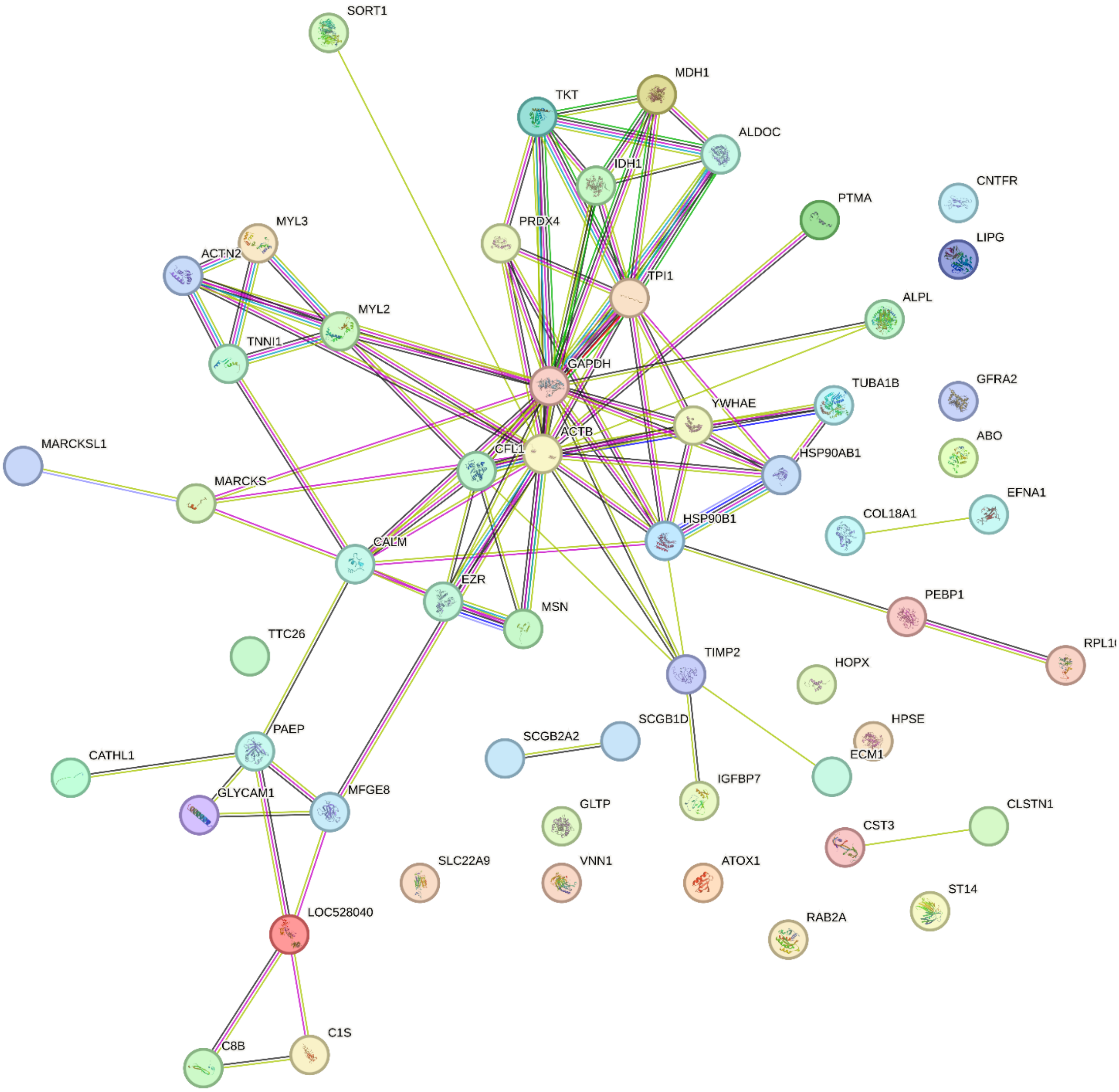

Supplementary Figure S1. STRING networks of the differential proteins observed at the beginning of the study (T0) in low-SCC milk from the conventional iodophor-based disinfectant (LSCC-C) group. The spheres represent proteins, while the lines represent the functional relationships between them (turquoise and purple: demonstrated; other colors, predicted). None of the proteins belonged to antimicrobial or immune defense functional classes or Uniprot Keywords. Protein codes are detailed in the Supplementary Dataset.

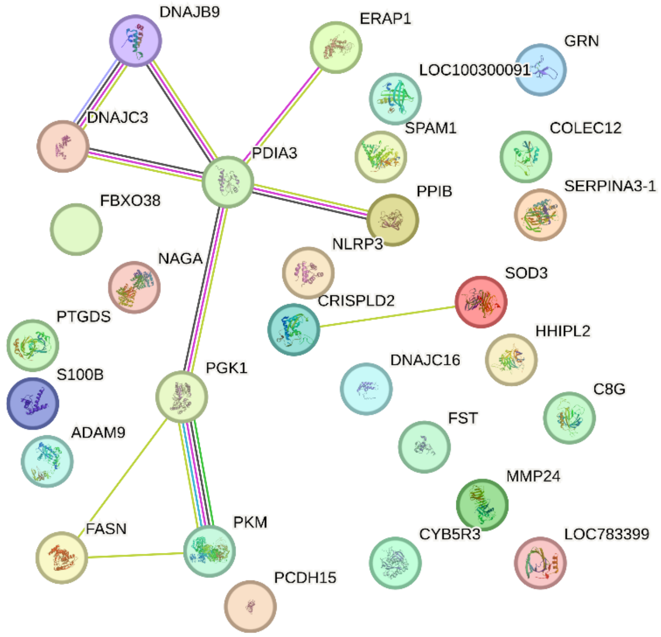

Supplementary Figure S2. STRING networks of the differential proteins observed at the beginning of the study (T0) in low-SCC milk from the *Lactococcus*-based disinfectant (LSCC-L) group. The spheres represent proteins, while the lines represent the functional relationships between them (turquoise and purple: demonstrated; other colors, predicted). None of the proteins belonged to antimicrobial or immune defense functional classes or Uniprot Keywords. Protein codes are detailed in the Supplementary Dataset.

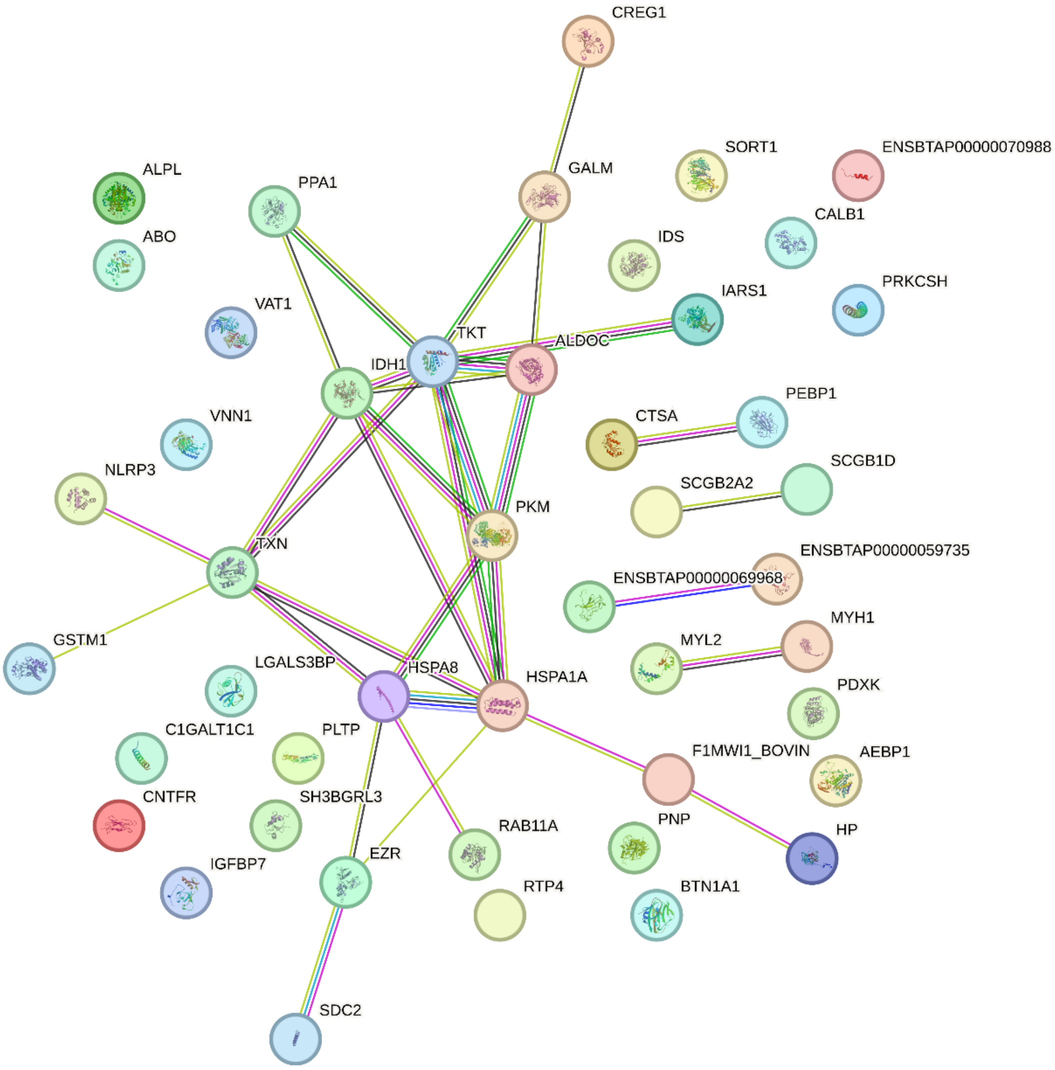

Supplementary Figure S3. STRING networks of the differential proteins observed at the end of the study (TF) in low-SCC milk from the conventional iodophor-based disinfectant (LSCC-C) group. The spheres represent proteins, while the lines represent the functional relationships between them (turquoise and purple: demonstrated; other colors, predicted). None of the proteins belonged to antimicrobial or immune defense functional classes or Uniprot Keywords. Protein codes are detailed in the Supplementary Dataset.

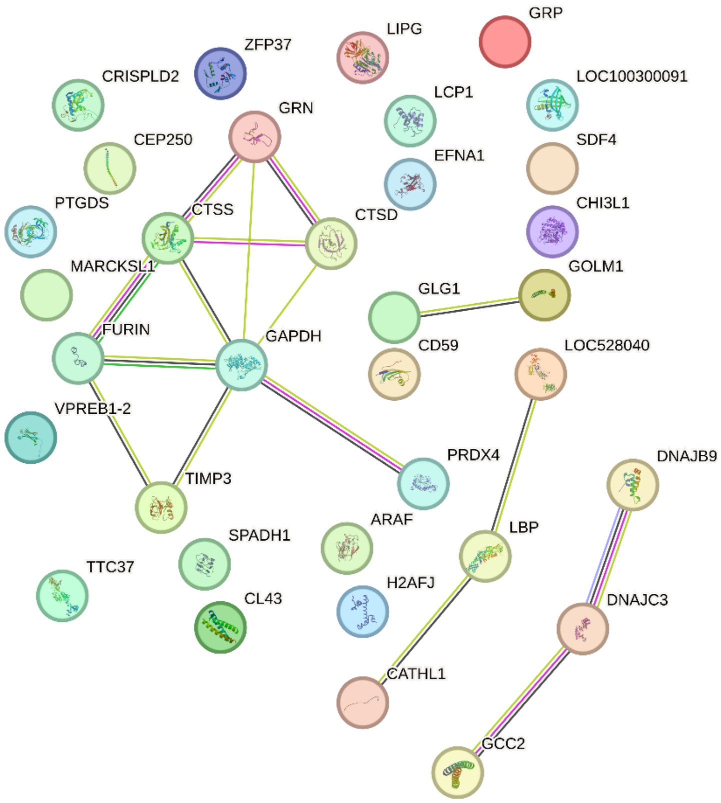

Supplementary Figure S4A. STRING networks of the differential proteins observed at the end of the study (TF) in low-SCC milk from the *Lactococcus*-based disinfectant (LSCC-L) group. The spheres represent proteins, while the lines represent the functional relationships between them (turquoise and purple: demonstrated; other colors, predicted). Protein codes are detailed in the Supplementary Dataset.

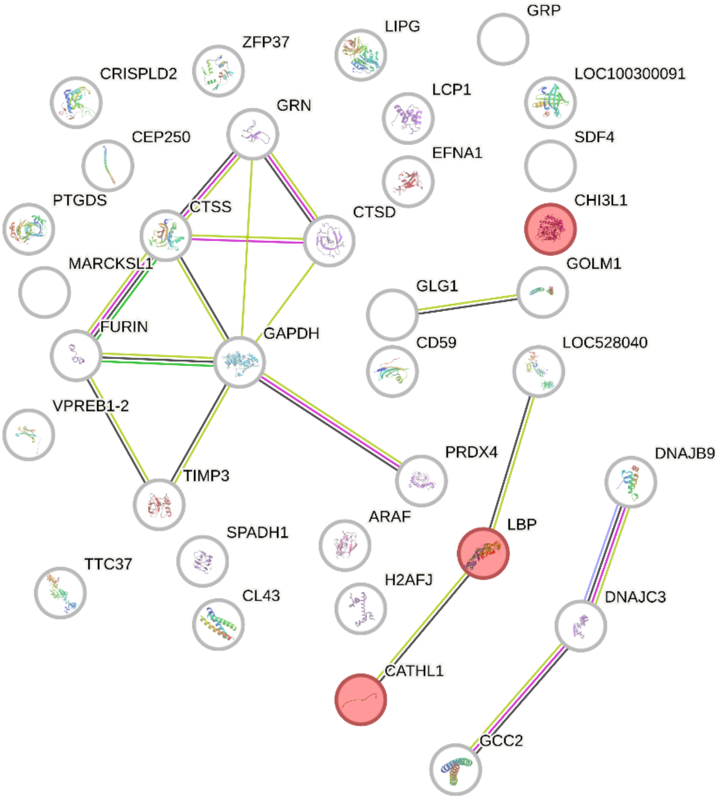

Supplementary Figure S4B. STRING networks of the differential proteins observed at the end of the study (TF) in low-SCC milk from the *Lactococcus*-based disinfectant (LSCC-L) group. The spheres represent proteins, while the lines represent the functional relationships between them (turquoise and purple: demonstrated; other colors, predicted). The proteins belonging to the Antimicrobial Uniprot Keyword are highlighted in salmon. Protein codes are detailed in the Supplementary Dataset.

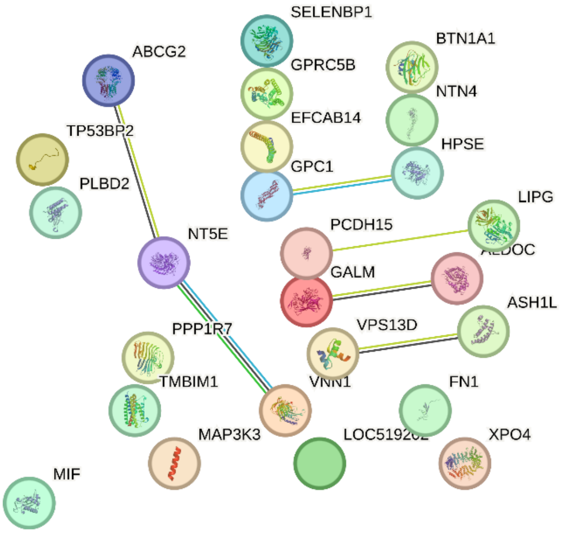

Supplementary Figure S5. STRING networks of the differential proteins observed at the beginning of the study (T0) in high-SCC milk from the conventional iodophor-based disinfectant (HSCC-C) group. The spheres represent proteins, while the lines represent the functional relationships between them (turquoise and purple: demonstrated; other colors, predicted). None of the proteins belonged to antimicrobial or immune defense functional classes or Uniprot Keywords. Protein codes are detailed in the Supplementary Dataset.

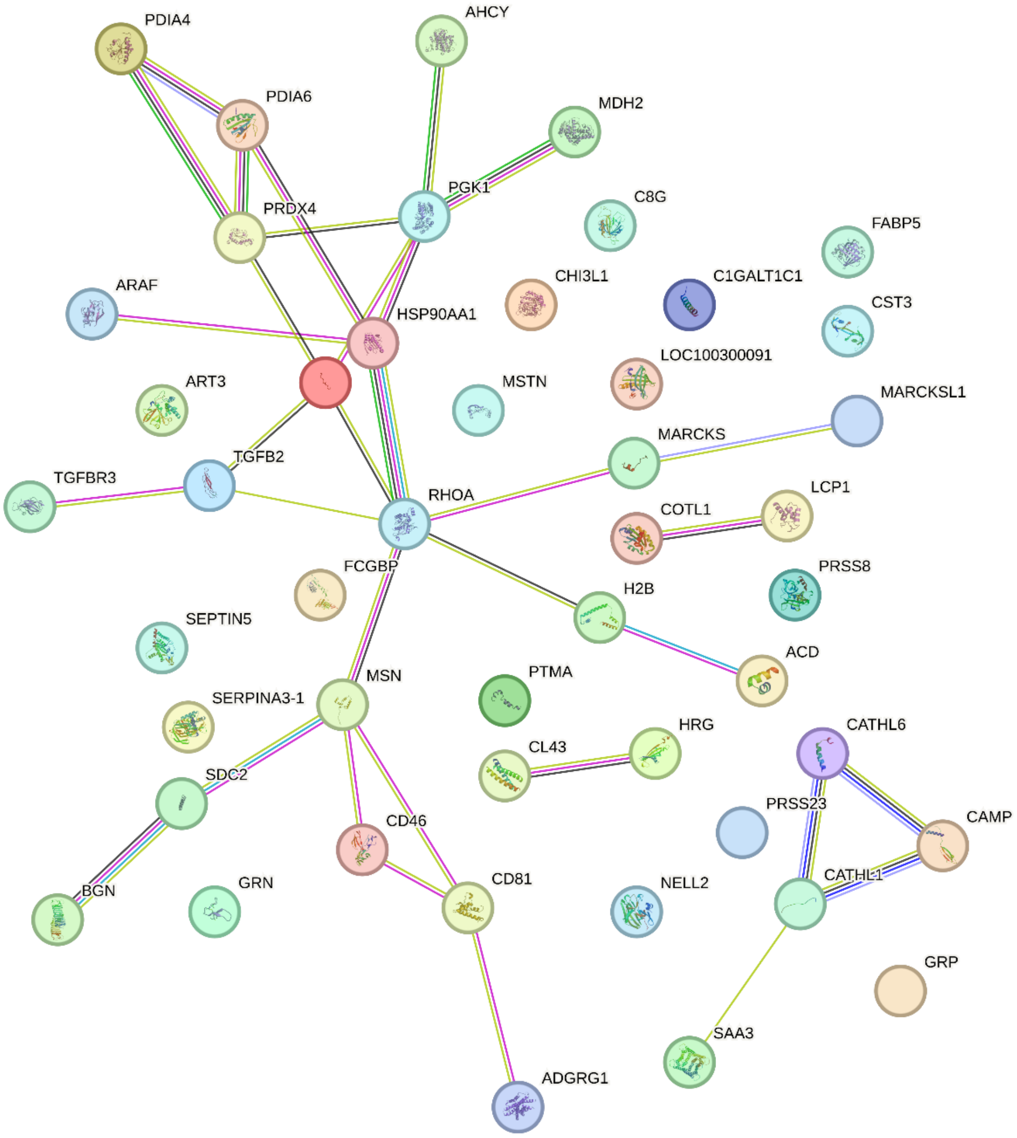

Supplementary Figure S6A. STRING networks of the differential proteins observed at the beginning of the study (T0) in high-SCC milk from the *Lactococcus*-based disinfectant (HSCC-L) group. The spheres represent proteins, while the lines represent the functional relationships between them (turquoise and purple: demonstrated; other colors, predicted). Protein codes are detailed in the Supplementary Dataset.

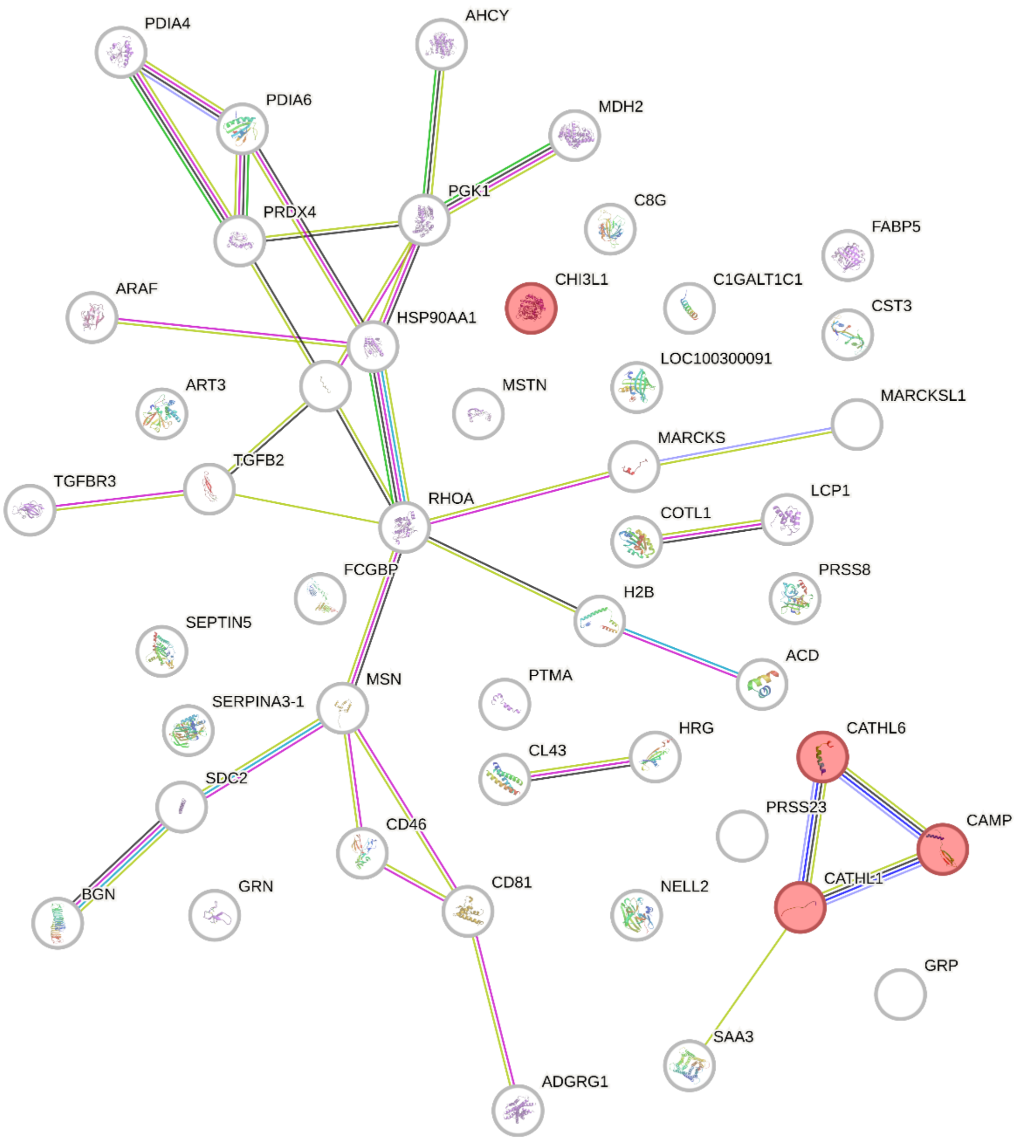

Supplementary Figure S6B. STRING networks of the differential proteins observed at the beginning of the study (T0) in high-SCC milk from the *Lactococcus*-based disinfectant (HSCC-L) group. The spheres represent proteins, while the lines represent the functional relationships between them (turquoise and purple: demonstrated; other colors, predicted). The proteins belonging to the Antimicrobial Uniprot Keyword are highlighted in salmon. Protein codes are detailed in the Supplementary Dataset.

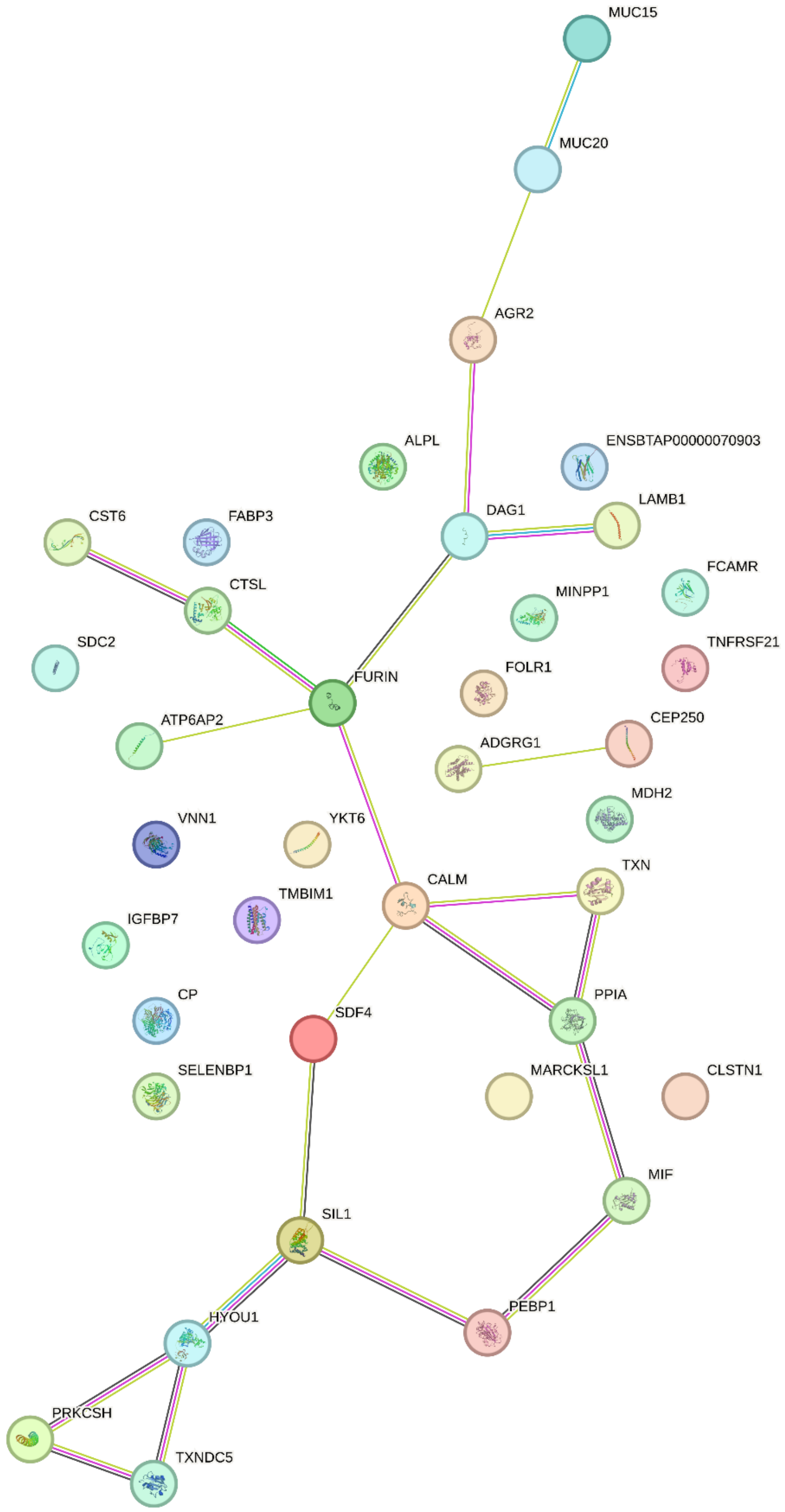

Supplementary Figure S7. STRING networks of the differential proteins observed at the end of the study (TF) in high-SCC milk from the conventional iodophor-based disinfectant (HSCC-C) group. The spheres represent proteins, while the lines represent the functional relationships between them (turquoise and purple: demonstrated; other colors, predicted). None of the proteins belonged to antimicrobial or immune defense functional classes or Uniprot Keywords. Protein codes are detailed in the Supplementary Dataset.

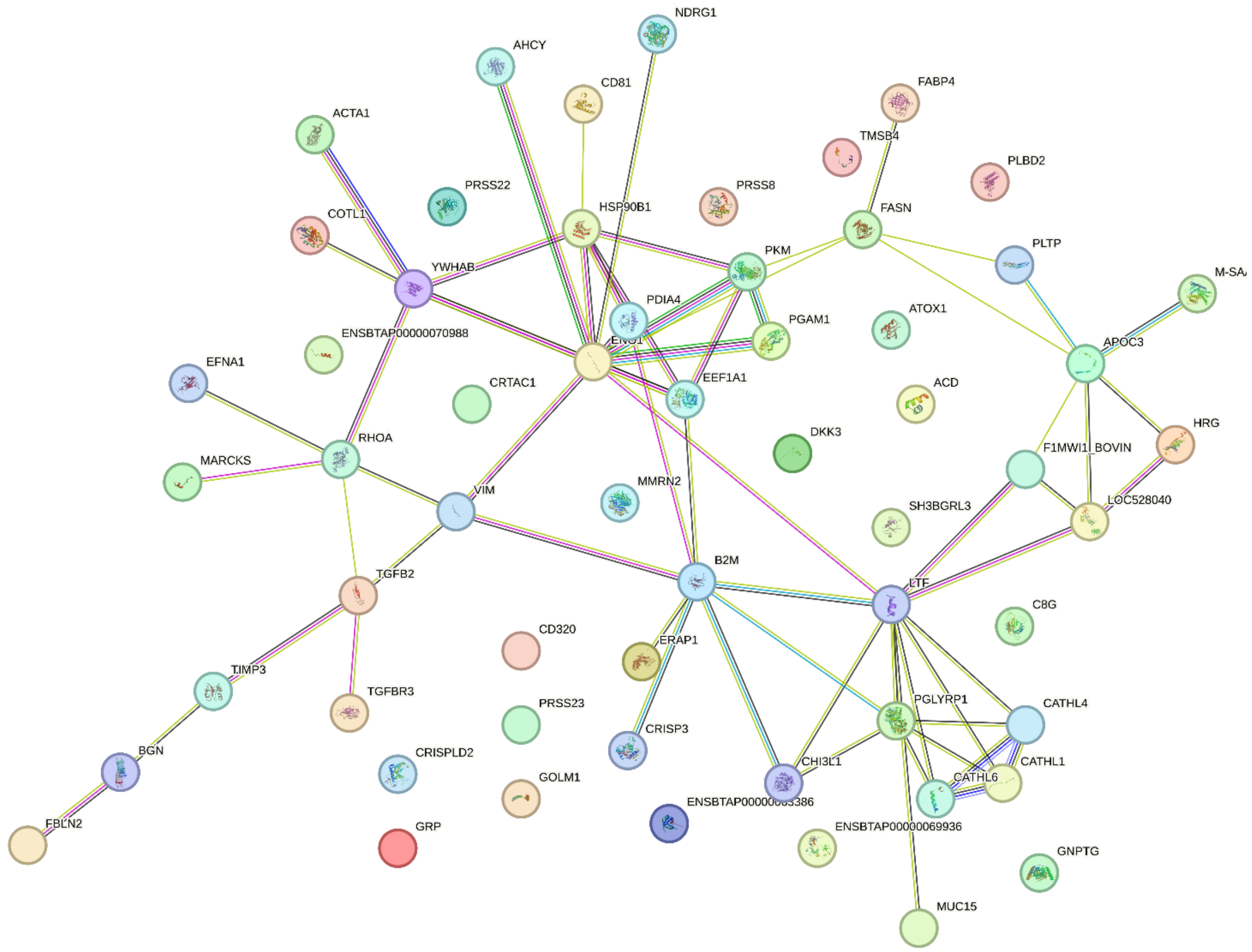

Supplementary Figure S8A. STRING networks of the differential proteins observed at the end of the study (TF) in high-SCC milk from the *Lactococcus*-based disinfectant (HSCC-L) group. The spheres represent proteins, while the lines represent the functional relationships between them (turquoise and purple: demonstrated; other colors, predicted). Protein codes are detailed in the Supplementary Dataset.

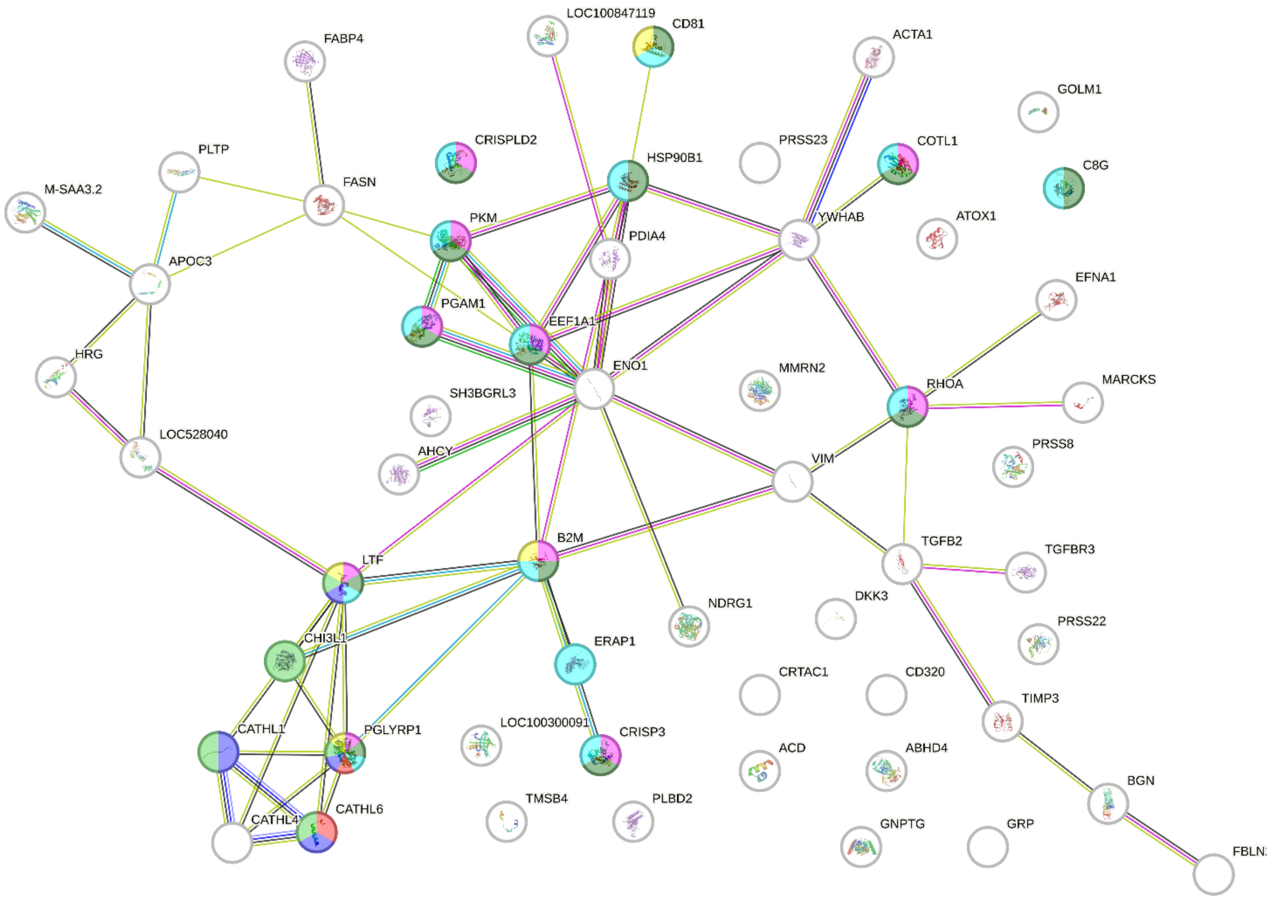

Supplementary Figure S8B. STRING networks of the differential proteins observed at the end of the study (TF) in high-SCC milk from the *Lactococcus*-based disinfectant (HSCC-L) group. The spheres represent proteins, while the lines represent the functional relationships between them (turquoise and purple: demonstrated; other colors, predicted). Protein codes are detailed in the Supplementary Dataset. The following Annotated Uniprot Keywords and Reactome Pathways are highlighted: Fungicide, salmon; Antibiotic, purple, Antimicrobial, green, Immunity, yellow; Neutrophil degranulation, pink; Innate Immune System, dark green; Immune system, light blue.
