# Supplementary material for: Impact of a teat disinfectant based on Lactococcus cremoris on the cow milk proteome
Source: BMC Vet Res. 2024 Oct 3;20:447. doi: 10.1186/s12917-024-04014-x (PMC11448288; doi:10.1186/s12917-024-04014-x)
Supplement: Supplementary file 3 — Supplementary Material 3 [file 12917_2024_4014_MOESM3_ESM.pdf]

Supplementary File 2. Original western immunoblotting results for the experiment summarized in Figure 7. Multiple exposure times of the same membrane are reported for Blot 1 (LSCC-C T0), Blot 2 (LSCC-L T0) and Blot 3 (HSCC-C and HSCC-L, T0 and TF).

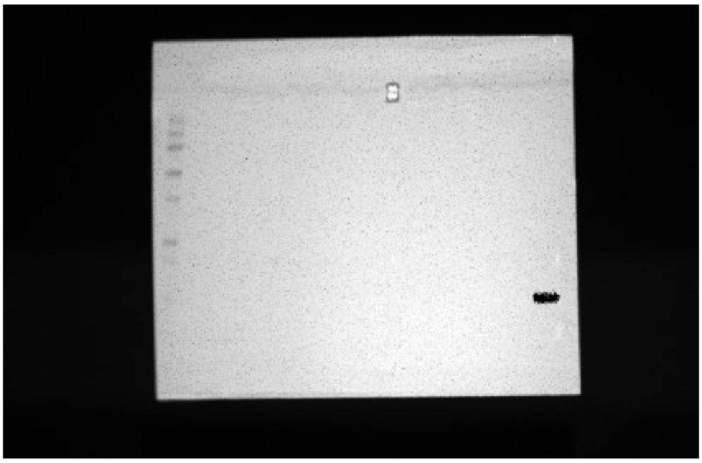

Blot 1, LSCC-C T0, 10 sec exposure

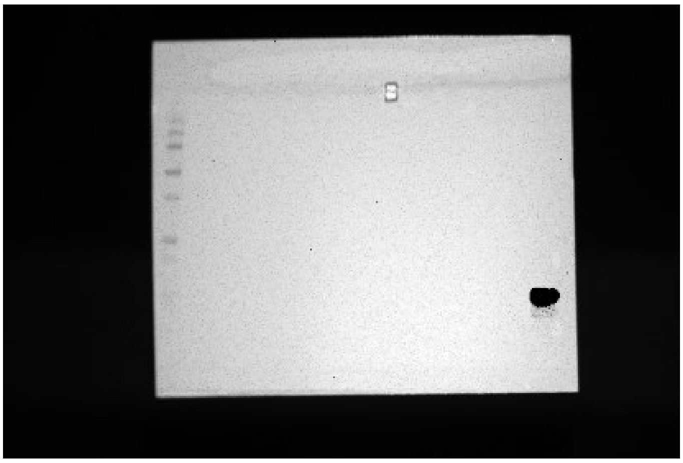

Blot 1, LSCC-C T0, 30 sec exposure

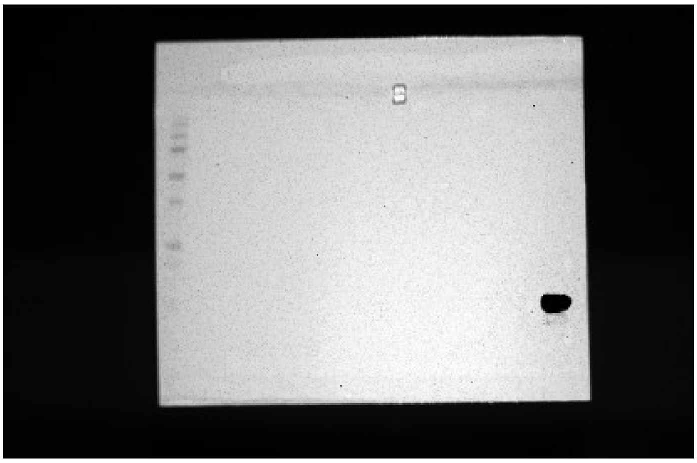

Blot 1, LSCC-C T0, 1 min exposure

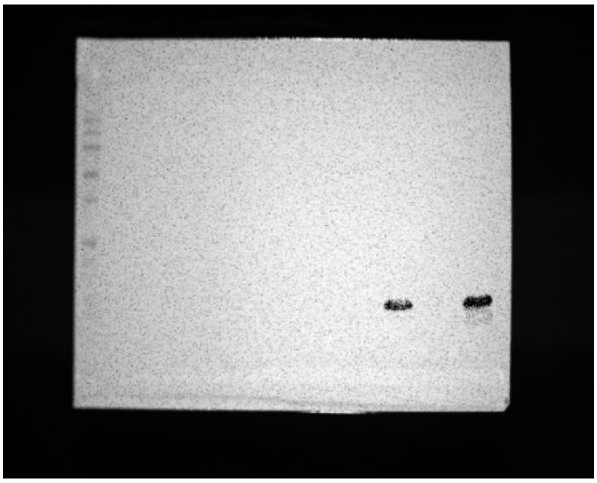

Blot 2, LSCC-L T0, 10 sec exposure

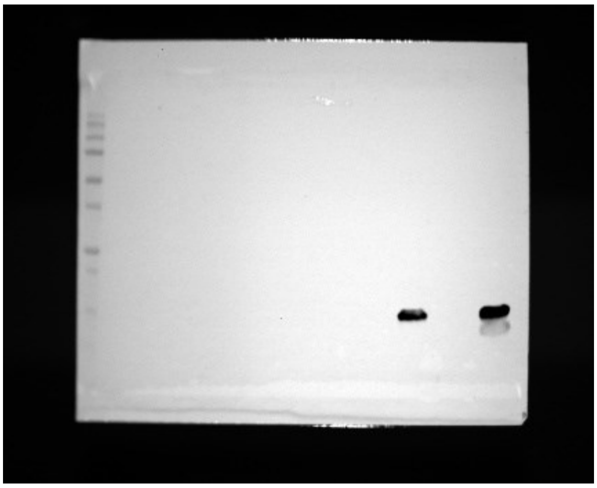

Blot 2, LSCC-L T0, 30 sec exposure

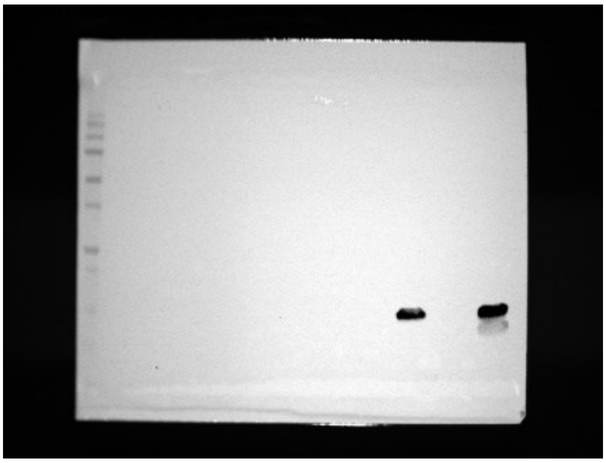

Blot 2, LSCC-L T0, 1 min exposure

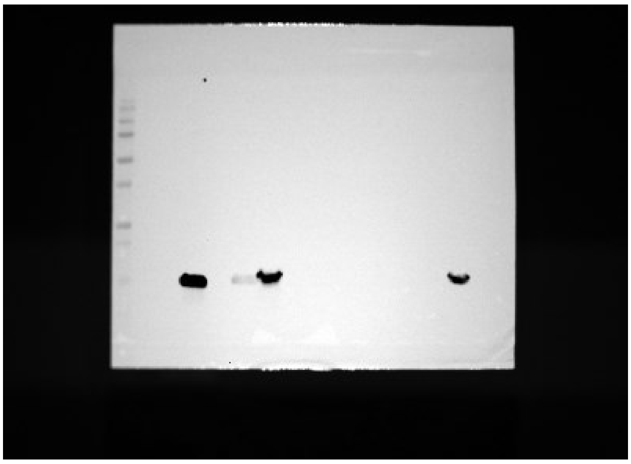
Blot 3, HSCC-C and HSCC-L TF, 30 sec exposure

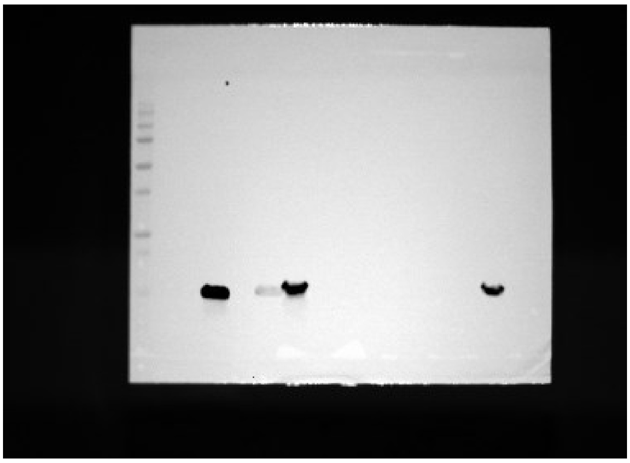
Blot 3, HSCC-C and HSCC-L TF, 1 min exposure

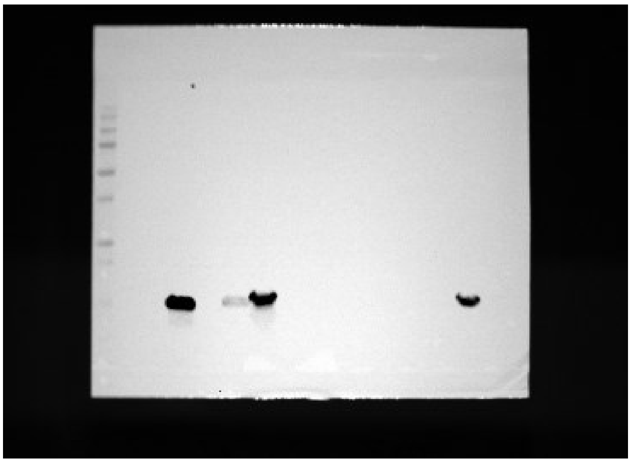
Blot 3, HSCC-C and HSCC-L TF, 2 min exposure
